# Interactions between a Heparin Trisaccharide Library and FGF-1 Analyzed by NMR Methods

**DOI:** 10.3390/ijms18061293

**Published:** 2017-06-17

**Authors:** María José García-Jiménez, Sergio Gil-Caballero, Ángeles Canales, Jesús Jiménez-Barbero, José L. de Paz, Pedro M. Nieto

**Affiliations:** 1 Glycosystems Laboratory, Instituto de Investigaciones Químicas (IIQ), Centro de Investigaciones Científicas Isla de La Cartuja, CSIC and Universidad de Sevilla, Américo Vespucio, 49, 41092 Sevilla, Spain; mariajose.garcia@iiq.csic.es (M.J.G.-J.); sergio.gil@iiq.csic.es (S.G.-C.); jlpaz@iiq.csic.es (J.L.d.P.); 2 Department Quim Organ 1, Fac CC Quim, Complutense University of Madrid, Avd Complutense S/N, E-28040 Madrid, Spain; ma.canales@quim.ucm.es; 3 CIC bioGUNE, Bizkaia Technology Park, Building 801A, 48170 Derio, Spain; jjbarbero@cicbiogune.es; 4 Basque Foundation for Science, Maria Diaz de Haro 13, 48009 Bilbao, Spain; 5 Department of Organic Chemistry II, Faculty of Science and Technology, University of the Basque Country, 48940 Leioa, Bizkaia, Spain

**Keywords:** NMR, FGF-1, STD-NMR, transient complexes

## Abstract

FGF-1 is a potent mitogen that, by interacting simultaneously with Heparan Sulfate Glycosaminoglycan HSGAG and the extracellular domains of its membrane receptor (FGFR), generates an intracellular signal that finally leads to cell division. The overall structure of the ternary complex Heparin:FGF-1:FGFR has been finally elucidated after some controversy and the interactions within the ternary complex have been deeply described. However, since the structure of the ternary complex was described, not much attention has been given to the molecular basis of the interaction between FGF-1 and the HSGAG. It is known that within the complex, the carbohydrate maintains the same helical structure of free heparin that leads to sulfate groups directed towards opposite directions along the molecular axis. The precise role of single individual interactions remains unclear, as sliding and/or rotating of the saccharide along the binding pocket are possibilities difficult to discard. The HSGAG binding pocket can be subdivided into two regions, the main one can accommodate a trisaccharide, while the other binds a disaccharide. We have studied and analyzed the interaction between FGF-1 and a library of trisaccharides by STD-NMR and selective longitudinal relaxation rates. The library of trisaccharides corresponds to the heparin backbone and it has been designed to interact with the main subsite of the protein.

## 1. Introduction

Fibroblast growth factor (FGF) signaling regulates mammalian development and metabolism, and its dysregulation is implicated in many inherited and acquired diseases, including cancer [1,2,3]. The key step for its activity is the assembly of a ternary FGF-FGFR-HSPG complex composed of FGF, its transmembrane receptor (FGFR) with intracellular tyrosine kinase activity, and heparan sulfate glycosaminoglycan (HSGAGs) [2,4,5,6]. This is a double trimeric complex (2:2:2) for FGF-1, in which all molecules are in contact with the other two types; the FGFR does this via two extracellular immunoglobulin domains. As a consequence of such supramolecular assembly, the intracellular domains of the two FGFRs approach and trigger intracellular autophosphorylation, leading to an enzymatic cascade towards the nucleus.

In this paper, we will focus on the interaction between the growth factor and the glycosaminoglycan. Now, it is commonly accepted that FGF-1 interacts with HSGAG through a positively charged swallow area [7,8]. In this region, two sub-zones can be differentiated. The largest region is capable of interacting with a trisaccharide through three sulfate groups, while the smaller is suitable for binding a disaccharide with two sulfate groups. The helical symmetry of the heparin backbone induces a good fit with both protein sites, while the negative charge distribution towards the opposite side, relative to the molecular axis, is also complementary [7,8]. From this observation, it was hypothesized that the main interaction subsite was interacting with a trisaccharide, while the other subsite reinforced the interaction by contacting a disaccharide from the same chain [7,9]. According to this analysis, the length needed for a complete interaction with FGF-1 is that of a hexasaccharide. By hexasaccharide we refer to a complete sequence made of non-unsaturated uronic acid at the non-reducing end, with a well-defined α/β stereochemistry at the reducing end. This definition is in contrast to that used when enzymatic depolymerisation fragments are used, where a 4–5 double-bond is created at the non-reducing end and an unprotected anomeric position mixture of anomers is generated at the reducing end. Several groups have been trying to find a specific interaction epitope without success, and it is now accepted that there is not a single specific sequence for the activation of FGF as selective as the interaction between Heparin and AT-III [10].

However, several sulfation patterns are known to interact preferentially in this region. Comparison of the activities of Heparan Sulfate (HS) oligosaccharide fractions derived from authentic HS containing FGF-activating as well as -nonactivating species revealed that the less sulfated HSs were less efficient activators [10,11,12]. An analysis of the FGF-1 mediated mitogenic activity of several heparin synthetic hexasaccharides and octasaccharides, capable of binding both sub-sites, was done by Martin-Lomas group, finding differences in activity that suggest that the sulfates opposite to the FGF-1 significantly modulated the activity [13,14,15]. Others have also used organic synthesis procedures for the preparation of di-, tetra-, hexa-, octa-, and decasaccharides [6,16,17,18] with the regular heparin substitution pattern. Furthermore, using solid phase synthesis, Seeberger and col. have accessed a number of compounds with the regular heparin structure, paving the way for new therapies [19].

The structure of the heparin regular region, 6SGlcNS—IdoA2S, corresponds to an extended helix with four residues per complete turn, leading to a two-fold axial symmetry characterized by almost opposite directions of the sulfate groups relative to the central axis. Similar structures have been observed in shorter oligosaccharides [20], down to trisaccharides [21].

**Figure 1:**
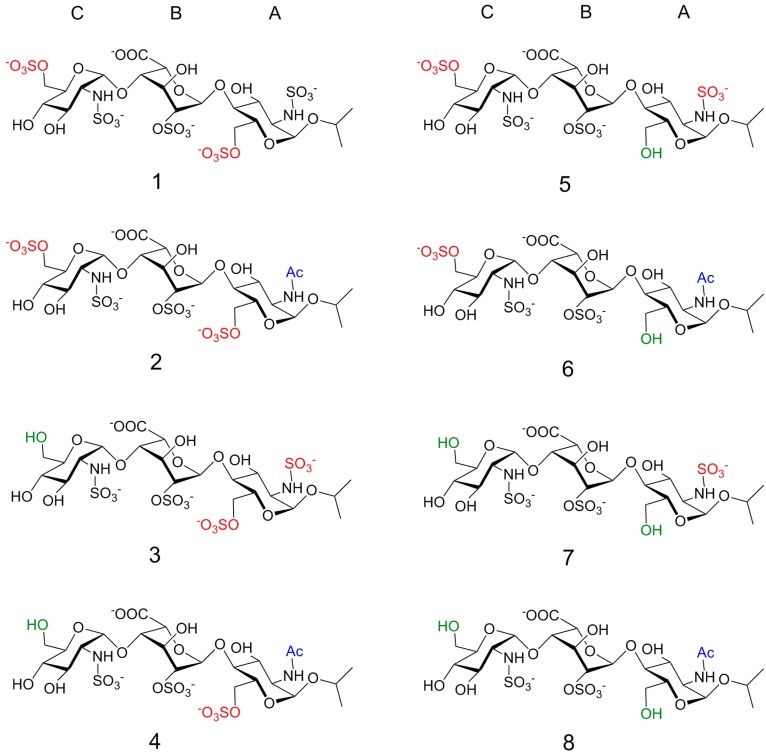
Library of heparin mimic trisaccharides. **1**–**4** were designed to interact with FGF-1, while **5**–**8** do not have the group 6-*O*-sulfate in glucosamine, and were designed to be inactive for the FGF-1—FGFR pathway, but active towards the FGF-2 pathway. We drew the trisaccharides formulae to represent the substituents relative directions in a three-dimensional space. The groups that are kept constant along the whole series are shown in black, and the sulfate groups that are subjected to permutation in red. Rings are labeled from the reducing end by letters A, B and C.

In order to analyze the molecular requirements of the primary interaction, we did synthesize a library of eight trisaccharides (Figure 1) [22]. Assuming the outcomes from the crystallographic studies and the helical symmetry of HSGAG [23], we designed trisaccharides with two invariant sulfate and sulfamate groups, while the rest of the positions sulfated in the heparin regular region were permuted in a combinatorial way [23]. According to previous studies, the invariant groups at positions C2 and B2 correspond to those needed for interaction with the FGF-2 surface in the larger subsite. Also, it is known that, for the interaction with FGF-1, an additional sulfate group is needed in A6. This it is present in compounds **1**–**4**, but is absent in **5**–**8** [24]. Then, according to our design, compounds of the **1**–**4** series are supposed to be able to interact with FGF-1, while the others, **5**–**8**, are not. In this work, we will quantify the interaction between the trisaccharide library and FGF-1, and we hope to provide valuable data about the role of the sulfate in position A6 in the selectivity between FGF-1 and FGF-2, and to identify if there is any additional selectivity or other role for the charges directed outwards from the binding interphase in the interaction between HSGAG with FGF-1. In a previous study, we analyzed the series of 3D structures; dynamic behavior and conformational properties relative to the iduronate conformational equilibrium of the free compounds, concluding that the whole series shares the same structural features, which are also the same as those for the HSGAG, and are therefore good models for exploring the role of the sulfation pattern in the interaction with proteins [22]. Previously, we studied the interactions between this library and a c-type lectin, Languerin, by STD, finding that the ligand structure remains upon binding [25]. In this work, we studied the interaction between the trisaccharide library and FGF-1, providing data about the role of the sulfate in position A6 in the selectivity between FGF-1 and FGF-2, and identifying any additional selectivity or other roles of the charges directed outwards from the binding interphase in the interaction with FGF-1.

Interaction between FGF-1 and charged synthetic sugars have been studied in several systems using a number of alternative techniques, including NMR [13,26,27,28], ITC, SPR [9], fluorescence polarization [29], and other biophysical methods. We would like to stress that, in this case, methods based on biological activity cannot be used, as a minimum length for the oligosaccharide larger than trisaccharide is necessary to ensure ternary complex formation, and, therefore, the activity. In addition, some of the biophysical methods, for example SPR, rely on competition experiments that, in this case, cannot be used because the available probes have a larger affinity for the FGF than the trisaccharides. In this case, then, methods that did not imply competition methods were needed.

Here, we report our results on the interaction between the trisaccharide library and FGF-1 by available ligand observed transient NMR methods, NOESY, STD [30,31], and T_1_, complemented with molecular modeling and docking calculations.

## 2. Results

### 2.1. NMR

**Figure 2:**
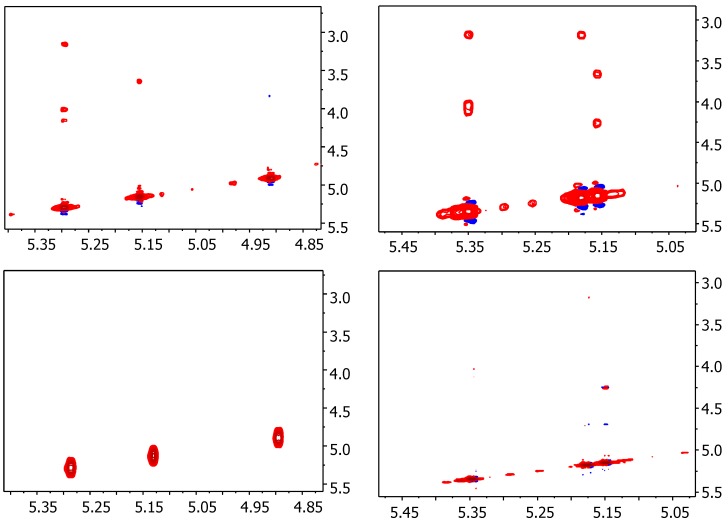
NOESY and transfer NOESY NMR experiments for **4** recorded for NOESY (**bottom left**), tr-NOESY (**top left**), and **1** NOESY (**bottom right**) and tr-NOESY (**top right**) recorded at 298 K at 600 MHz. Display parameters, low contour level, number of contour levels, and separation between them are the same of all four experiments. Units are in ppm.

First, we assessed the binding of trisaccharides to FGF-1 by evaluation of the NOESY in the presence of FGF-1 by a shift of the correlation time by complexation towards the spin diffusion limit (negative NOE) [32]. At room temperature at 600 MHz, the cross-peaks for free trisaccharides **1** and **4** were extremely weak or null; while in presence of FGF-1 and in the same conditions, they correspond to neatly positive NOE effects (Figure 2).

**Figure 3:**
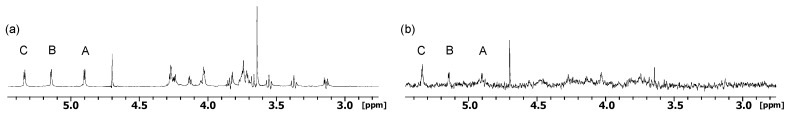
NMR ^1^H (**a**) and STD-NMR (**b**) experiment recorded at 600 MHz for **4**, 298 K, see experimental part for details. Peaks for the anomeric of ring A, B and C are marked.

**Figure 4:**
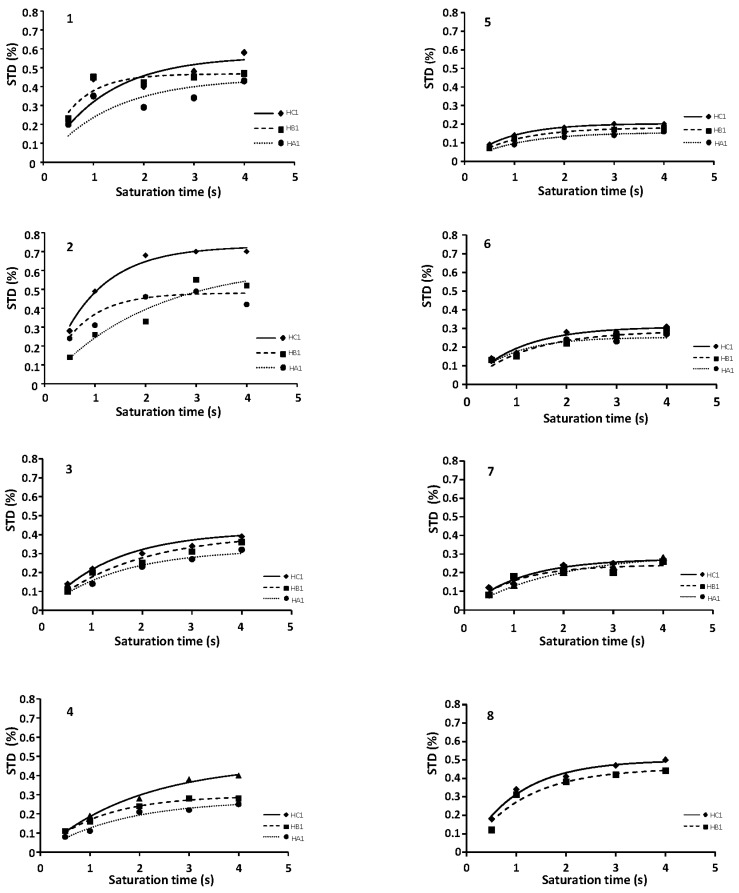
STD-NMR growing curves for **1**–**8** recorded at 600 MHz, 298 K. See experimental part for details.

STD-NMR [30] studies were acquired for all the trisaccharides in the library in presence of FGF-1 using the same experimental conditions (see material and methods). Transference of saturation for all trisaccharides probed the interaction with the protein. Nevertheless, in contrast to the case of Langerin interaction [25], the results differed between the different compounds, indicating the existence of some differences in binding. Unfortunately, the spectral crowding only allowed us to accurately integrate the three anomeric signals along the saturation times, see Figure 3. Using a methodology previously described for disconnect the effect from proton relaxation on the STD values at a given time, the values of the initial rate of growth of STD (STD_0_) for the considered signals were obtained for the signals under consideration (Figure 4, Table 1) [33]. The distribution of magnetization along the trisaccharide chain is rather homogeneous, and is compatible with an extended interaction along the positive cleft of FGF-1, where the main binding site is located. These results contrast with those obtained for the binding to Langerin using the same library of compounds where the binding was at the non-reducing end, and the magnetization strongly decays along the chain towards the isopropyl moiety [33].

For the analysis, we distinguished two series of trisaccharides: those with 6-*O*-sulfonate at the reducing end residue (A), series 1, which were supposed to activate the FGF-1 pathway; and those that did not have it, series 2, which were assumed not to activate the FGF-1 [34]. We confirmed this, as the binding to FGF-1 was stronger for series 1 than for series 2 when the same substitution patterns were compared (**1** with **5**, **2** with **6**, **3** with **7** and **4** with **8**).

It has been proposed that the presence of a sulfate in position 6 of the glucosamine at the reducing end of a trisaccharide that interacts with the main binding site is essential for the differentiation between FGF-1 and FGF-2 [35]. Our results are consistent with a lower interaction between FGF-1 and those trisaccharides without this sulfate (see Figure 4 compounds **5**–**8**). However, we want to stress that there is still some interaction, in spite of the absence of this group.

When curves for trisaccharides **1** and **4** are compared, **1** exhibits a larger STD value than **4**. As they have the same distribution of binding groups at the interphase with the protein and the structure of the complex should be the same for both, this result can be explained assuming that **1** uses an extra binding mode to interact with FGF-1. We propose that it should be similar to that observed in the dimeric crystallographic structure, and **1** uses the extra charges at the upper face absent in **4**. We obtained similar results when we evaluated the interaction with FGF-1 of five hexasaccharides by SPR [9]. The largest binder was the compound with additional sulfate groups pointing outwards **1** [9]. Additionally, these results also ranked the relative interaction of the peripheral groups. When the binding curves for **2** and **3** are compared, the later has a similar binding to **4**, while the other is larger, similar to **1**, highlighting the greater influence of the group at position 6 of residue C present in **2**. Unfortunately, we cannot explain the slightly larger binding of **2**, compared with **1**.

**Figure 5:**
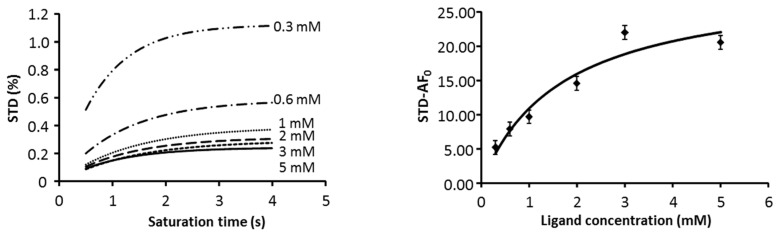
Estimation of IC_50_ for **1** by STD-NMR growing curves recorded at 600 MHz, 298 K. Plot of the STD growing rate at different ligand concentrations for the calculation of STD_0_ from the initial rate of STD (**left**). Plot of the STD_0_-AF at different ligand concentrations of **1** for the calculation of IC_50_ (**right**), see experimental part for details.

**Figure 6:**
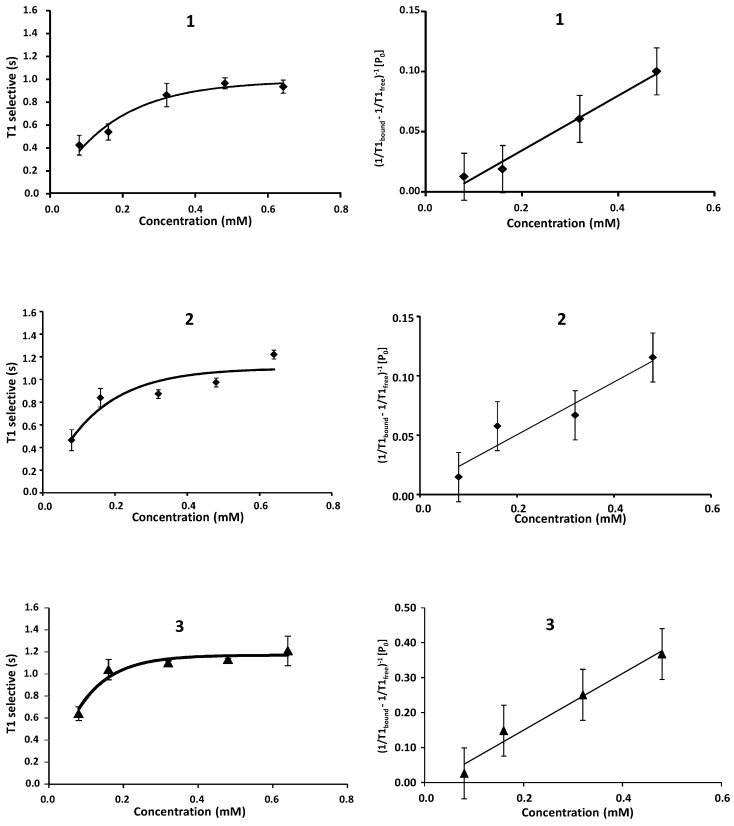

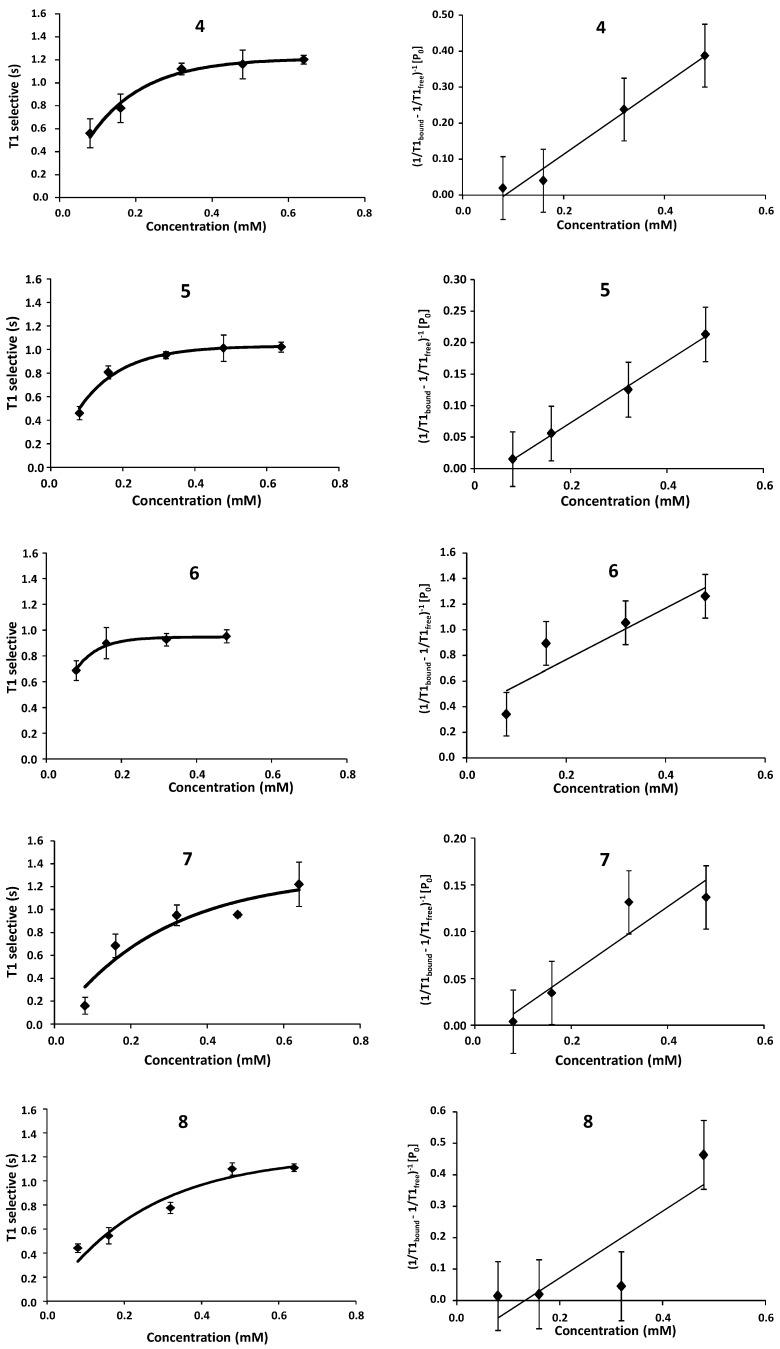
IC_50_ calculation using T_1sel_ method **1**–**8**.

IC_50_ values using STD-NMR were calculated for compound **1** at different concentrations based on the initial rates of the affinity factor method (Figure 5) [33]. Although an IC_50_ of 1.70 ± 0.01 mM was obtained, we cannot support the validity of the result due to several drawbacks of the method. In this case, due to the electrostatic nature of the complex, the gap between the protein and the ligand protons is very large, yielding low magnetization transfer values. On other hand, the small binding constant implies a large consumption of trisaccharide due to the high concentration needed to complete a meaningful titration curve. This, together with the large amount of time needed to record the full series of experiments, forced us to consider another method for the estimation of the IC_50_. Thus, we explored the IC_50_ calculation using selective T_1_ at different concentrations (Figure 6) [36]. The results are considerably smaller that those obtained by STD_0_-AF, see Table 2. Nevertheless, they are in agreement with the values obtained by Yu-Peng et al., when performing the same measure with disaccharides and FGF-2 by ITC [18].

The results showed a high dispersion, probably reflecting potential extra modes of binding and additional sites of interaction. However, the values for **1**–**4**—those with 6-*O*-sulfate in the reducing end glucosamine—were always lower, indicating a large interaction.

### 2.2. Docking

Experimental interaction NMR data were complemented with a computational analysis. This was based on previous findings on the interaction between FGF-1 and heparin oligosaccharides [7,8], where the three-dimensional structure of the heparin was not modified upon binding, conserving its characteristic helical arrangement with four residues per turn [23]. Previous analysis of the interaction models showed that the FGF-1 binding region can be considered to be divided into two subsites. The main one is where a trisaccharide GlcN-IdoA-GlcN interacts with the protein, establishing three charge–charge interactions involving the sulfates of the same side. The secondary site is in an adjacent region, and contributes to the binding by interacting with a 2-SO_3_-IdoA-6-SO_3_-GlcN sulfated disaccharide through the contact of two sulfates groups [7]. Additionally, a previous study using NMR and MD describes that all free trisaccharides from the library conserve the same helical structure as heparin [22].

We focus our interest on the space occupied by the trisaccharide in the main binding subsite, and docking calculations were performed with the starting models in this region of the FGF-1 using GLIDE, as implemented in Maestro [37]. The influence of the non-participant charged groups in the structure and in the strength of the complex was considered. Details are in the experimental part and in the supplementary material.

We compared the results obtained for **1**–**4** and for **5**–**8**. **1**–**4** correspond to the trisaccharides with the essential sulfate group for FGF-1 interaction [22]. In general, **1**–**4** bind into the main binding groove, while **5**–**8** poses tend towards the alternative site, but they are very disordered. When the affinities were compared pairwise, they showed a general decrease in interaction strength for the non-sulfated in position 6 of GlcN A.

**Figure 7:**
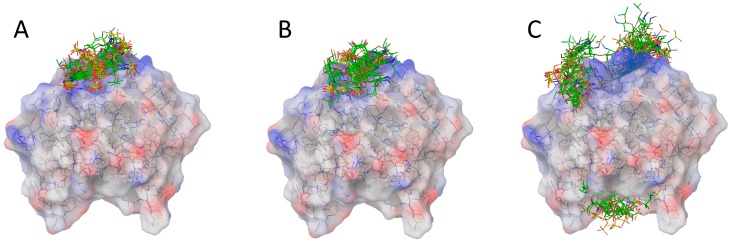
Best docking structures for **1** (**A**); **4** (**B**) and **8** (**C**). Colors in the surface of the proteins are according the electrostatic potential: Red negative and blue positive.

Regarding the first series, the best and simplest result was obtained for **4**. **4** has only the three sulfate groups needed to interact in the main binding site, and only a potential binding mode was possible (Figure 7). This corresponds to the same site and pose as that described experimentally by both X-ray and NMR [7,8]. In contrast, **1**, which has additional sulfate groups in the external side, showed poses corresponding to a 180° rotation along the molecular axis similar to those found for the crystallographic structure of the dimer AMX [7]. In the same conditions, **3** behaves similarly to **4**, and **2** to **1**. From this observation, the importance of the sulfate in position 6-*O* of ring C in the interaction with FGF-1 is implied. The poses obtained for **4** are in agreement with the experimental data, and reproduce well the experimental structures. The **3** case is similar, but some differences can be found at the level of residue A. This residue is rotated towards the protein to compensate for the additional charge. In the results for **1** and **2**, with an additional sulfate group in ring C directed outwards, we observed more structures generated from changes in direction along the binding site and/or in the face of the carbohydrate that interact with FGF-1. In some cases, a rotation between ring B and A towards an anti-disposition was observed, while rings C and B interacted with the protein in the same manner as **4**. In addition, it can be observed that when one of the rings, particularly A, has two sulfate groups, it tends to rotate relative to the protein lying in the surface. We suppose that this arrangement over the protein yields a better charge compensation (see Figure 7 for **1** complexed with FGF-1). However, the NMR data are not in agreement with such a possibility, and all of them keep the heparin structure without modification in the glycosidic linkages.

**Figure 8:**
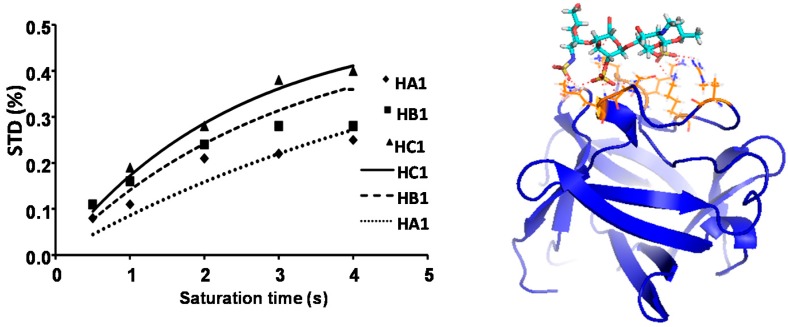
Representation of STD experimental values (dots) vs. theoretical ones calculated with CORCEMA-ST (lines) from the best structure calculated by docking (**right**) for compound **4**. Protein secondary structure elements are shown in blue ribbons, trisaccharide **4** is shown in sticks color coded for the elements and in orange are the side chains of the protein in close contact with the ligand **4**.

On the other hand, most of the results for compounds **5**–**8** were far from the main binding site proposed from the analysis of the crystallographic structures for the complex with longer saccharides (pdb: AMX), confirming the importance of the sulfate at ring A for the interaction with FGF-1 [24]. Here, the dispersion of binding modes and orientations is larger accused. For instance, one of the most frequent structures found is one in which the glycosidic linkage between ring A and B is in anti-disposition. We interpret this unrealistic structure as a way of compensating for the loss of the 6-sulfate group in ring A using the interaction of the charged sulfonamide, see results for **5** and **7** in the supplementary material. When we performed the docking calculations using a larger grid, we obtained additional binding poses at a secondary subsite for 2-SO_3_-IdoA-6-SO_3_-GlcN disaccharide, as well at alternative sites far from the main binding place. These sites were previously described by Ornitz (see Figure 7) [34]. Interestingly, in the case of **8**, one site on the opposite face of the FGF-1 from the main binding site was particularly well populated (see Figure 8).

When the same grid was applied to compounds **1**–**4**, we obtained docking structures where the trisaccharides also interacted with the secondary site. But in all cases, this was as a minor mode. However, no solutions were detected at the opposite site to the degree that they were for **8**, which could be reflect the existence of a different real binding when only two sulfate groups are present in the molecule.

### 2.3. CORCEMA

The complete relaxation and conformational exchange matrix (CORCEMA-ST) [38] method was used to analyze STD-NMR results using the lowest energy single conformation obtained by docking, using GLYDE [37] as model. Unfortunately, the experimental data were not accurate enough to perform a deeper quantitative analysis [39]. Only the results from **3** and **4** could be adjusted to a single binding mode using a single structure based on the crystallographic data (see Figure 8 and supplementary material). We believe that the other alternative structures for the rest of the trisaccharides may interact with the additional sulfates in the opposite face and that the STD results are an average of both poses and therefore cannot properly be described.

## 3. Discussion

A comparative study of the binding of a library of trisaccharides to FGF-1 has been completed. The library was based on the structure of heparan sulfate and designed to analyze the potential role of non-participating charged groups. NOESY experiments on the complex revealed that all the trisaccharides retain the same 3D structure as in the free state, and that this is compatible with the heparin helical structure [23]. STD data for the whole library are in agreement with an extended binding mode where the magnetization is distributed along the ligand.

When growing STD curves were analyzed, differences on the binding strength could be observed. This indicates a different recognition mechanism from that found in Langerin using the same library [25]. Binding constants and STD curves indicated that the presence of a sulfate group in position 6 of the reducing end glucosamine is relevant for the interaction, as most of the trisaccharides without this key element, **5**–**7**, show a decreased interaction with FGF-1. When the results were combined with docking results, the binding place for the trisaccharides with 6 *O*-sulfate at the A ring could be determined, and corresponded to the main binding site found on X-ray structures. The importance of the sulfate at this position has been previously proposed for FGF-1/FGF-2 selectivity [24]. 

In some cases, alternative binding modes can be proposed. Trisaccharide **4** has the minimum sulfation pattern for efficiently interacting with FGF-1, while **1** has additional sulfate groups in the upper face. This makes **1** suitable for an additional binding mode corresponding to reverse (up-down) binding. Logically, **1** has a larger affinity than **4**, as it can be bound using two modes. The similitudes of binding modes and STD curves between **1** and **2**, and **4** and **3**, suggest a different influence on the binding by the sulfate in position 6 of ring C from that in position N of ring A.

In order to better understand the reasons behind this behavior, docking and CORCEMA-ST calculations were performed [40]. Unfortunately, when we tried to apply CORCEMA-ST to the docked structures, we could only fit two of the obtained structures—**3** and **4**—to a single binding mode. Their binding modes are superimposable with the X-ray structure. The others two trisaccharides—**1** and **2**—with sulfate in ring C showed a larger affinity, belonging to the same series. We propose that is due to a second binding mode in the same site, consistent with rotation around the molecular axis, as can be seen in the crystallographic structure of the complex with a heparin hexasaccharide [7]. Therefore CORCEMA-ST cannot be applied in this case, and there are not enough data for a deeper analysis that can consider several poses within the binding site.

We have observed in docking calculations that modes arising from the exchange between both ends and sides can be detected for **1** and **2**. They are analogous to X-ray and NMR structures reported previously. We also performed docking calculations with the trisaccharide series with no sulfate at the 6 position of ring A. They give reasonable results when an extended site that included a whole FGF-1 was used; they also interact in the second interaction subsite proposed for two sulfate groups. Also, as in some cases, such as **8**, they interact out from the proposed binding sites.

We first calculated the binding constant for trisaccharide 4 using the STD_0_ methodology, but the value was unexpectedly weak, at 1.7 mM, and the amount of compound and time needed was excessive. Then, we calculated the binding strength using the T_1sel_ methodology, and the IC_50_ are in the uM range. 

## 4. Materials and Methods

Synthesis of compounds **1**–**8** was previously described [22]. NMR experiments were recorded in a BRUKER 600 MHz AVANCE III HD with an ASCEND magnet equipped with a QCI S3 H/F-C/N-D 05-Z cryoprobe.

Human recombinant FGF-1 consisting of 141 residues expressed in *E. coli* was purchased from Peprotech.

*STD-NMR Experiments.* All the samples were prepared in 250 μL 5 mM phostate buffer at pH 6.0 and 100 mM NaCl in 99.9% D_2_O. The concentration of the protein was 19 µM, and the ligand was 1.6 mM. The ratio between the protein and ligand was 1:84. The experiments were performed at 289 K. STD-NMR experiments were carried out with spin-lock to suppress protein signals. Moreover, we tried to suppress the solvent using excitation sculpting with gradients, and we used a shaped pulse train for saturation, alternating between on and off resonance. The on-resonance and off-resonance frequencies were set to 0.7 and 40 ppm, respectively. A spoil sequence was used to destroy unwanted magnetization. The saturation times were 0.5, 1, 2, 3 and 4 s, with an additional delay set to 3.6, 3.1, 2.1, 1.1 and 0.1, respectively, in order to keep the relaxation delay constant at 4.01 s for all experiments. STD NMR experiments were performed with 2048 scans with a total experimental time for every point of 5 h.

The STD initial rates (STD_0_) [41] were calculated from the curve of STD vs. saturation time. To do so, we utilized the equation: STD_0_(*t*) = a [1 − exp (−bt)], where a represents the asymptotic maximum of the STD build-up curve (STD_max_), b is a rate constant that depends on the relaxation properties of a given proton that measures the rate of the STD build-up (*K*_sat_), and *t* is the saturation time (*t*_sat_). The STD_0_ values were obtained as the product of the ab coefficients [41].

IC_50_ was obtained using the initial rates of the STD affinity factor approximation. The STD-AF initial slopes (STD-AF_0_) were calculated as before from the curve of STD-AF vs. saturation time at several ligand concentrations. From these initial slopes, the binding isotherm was built (STD-AF0 vs. ligand concentration), and the curve fitted to a Langmuir equation, from which the IC_50_ value was determined [33].

*Selective T*_1_ *NMR experiments.* The samples were prepared using the same buffer at 20 μM protein concentration, while ligand concentration was 80, 160, 320, 480 and 640 μM. The ratios between protein and ligand were then 1:4, 1:8, 1:16, 1:24 and 1:32, respectively. We carry out studies on ^1^H longitudinal relaxation rate (1/T_1_(sel). The relaxation times were measured by inversion recovery pulse sequence.

To determine the IC_50_, we adjusted the experimental values of the intensity signals vs. recovery delays to obtain an exponential function. We obtained the selective T_1_ value by f(*t*) = I_0_ × [1 − a × exp (−t/T1)]. In a ligand-receptor system where the ligand is present in large excess over the receptor binding sites, the ligand relaxation rate is describe by 1/T_1_obs = 1/T_1_free + fbound/(T_1_bound + tbound), where f_bound_ is the fraction bound ([PL]/[L]) and τ_bound_ is the lifetime of the bound state. The relationship between total ligand concentration and T_1_ is then [P]_0_T_1obs_ = ([L]_0_ + IC_50_)(T_1bound_ + τ_bound_), and a plot of [P]_0_ T_1obs_ versus [L]_0_ gives a straight line with IC_50_ obtained from the intercept of the *x* axis [42].

*Molecular Docking.* The protein databank structure 2erm was used for the studies of docking performed with Glide [37]. Each trisaccharide was built by modifying the hexasaccharide of the 2erm structure. We prepared our model by superposition of the reducing end of the trisaccharide with the non-reducing end of the 2erm complexes aligned with the backbone of the trisaccharide. Then, we performed a conformational search with Iduronic Acid restraints, obtaining several structures of the ligand. Then, Simple Docking was run. To do so, the grid was first generated by defining the centroid of the ligand as the center of the box, and a box size of 16 Angstrom was set. Then, a flexible docking standard precision was carried out without sampling the conformation of the rings, while penalizing the non-planar conformation of the amide bonds. Forty poses were minimized, finally resulting in at least 20 poses (see supplementary material).

*STD theoretical simulation: CORCEMA-ST* [43]. Of the 20 poses of the complex obtained by flexible docking calculations, we chose the poses with the sulfate groups oriented to protein. They were used to obtain the STD theoretical values. Hence, the conditions for obtaining these values were: the concentration of protein was 30 uM, the concentration of ligand was 1.5 mM, the correlation times of protein and ligand were 22 and 0.4 ns, respectively. The constant of dissociation was 10 μM, a cut off of 8 A from the ligand and 10 ps for the internal correlation time of methyl groups.

## 5. Conclusions

The FGF-1 binding site for HSGAG can be subdivided into two adjacent regions: one that interacts with a trisaccharide and another that interacts with a disaccharide, corresponding to two adjacent positively charged patches. In this work, we have analyzed the interaction between a library of trisaccharide synthetic HSGAs with the same internal scaffold but different sulfation patterns, which was designed to analyze the influence of the substitution pattern in the molecular interaction with FGF-1 (Figure 1). The design assumed that the interaction occurs mostly in the main site, where three sulfates can interact simultaneously.

As expected, the transfer NOESY data indicated that the geometry of the trisaccharides is not modified upon binding, and is similar to the free state: linear and helicoidal with the sulfate groups pointed in two opposite orientations relative to the main axis of the saccharide, and one side directed to the FGF surface. This arrangement is compatible with the structures in solutions of complexes with hexa- and octasaccharides and FGF-1 [8].

STD-NMR results were compatible with an extended binding. The STD values were small, reflecting the fact that binding occurs through a large gap of non-protonated side-chains that are difficult to magnetically diffuse. After an attempt to calculate the IC_50_ using an STD method, we used selective T_1_, obtaining better results. The values of the interaction obtained verify that the FGF-1 interaction is better when a sulfate group in the reducing end of the trisaccharide is participating.

The results of **1**–**4**, the series with 6-*O*-sulfate in ring A, are compatible with the interaction described for the oligosaccharide chain in the larger binding site using the three sulfate groups to interact. The IC_50_ values are compatible with an additional binding mode for **1** and **2** that holds sulfate groups on the external side. Estimations of STD using CORCEMA-ST for **3** and **4** agree with a single binding mode as predicted by docking calculations with GLIDE, but that failed when we tried to calculate the STD for **1** and **2** CORCEMA-ST using a single structure. The compounds without this sulfate, **5**–**8**, showed a worse affinity in all cases. The docking results for **5**–**8** are compatible with an interaction mostly in the alternative sub-binding site, probably better suited for a two-charge interaction. In addition, the trisaccharide **8** docked structures at the southern part of FGF-1 were consistent with previous works by Ornitz [34]. The STD results exposed here demonstrate that a sulfate in position 6 of the glucosamine at the reducing end is required for a complete interaction in the main binding site of FGF-1 [1].

During the elaboration of this paper, we realized that as the length of the GAG decreases, it can bind to more sites than the longer homologues. We can speculate that the selectivity necessary for biological regulation is obtained by adding modules to the linear oligosaccharide chain that have a particular pattern, and which, by adding addition interactions, can tailor the selection.

## Figures and Tables

**Table 1:** STD_0_ and relative STD_0_ for **1**–**8**.

Compound	STD_0_			Relative STD_0_		
	H1-A	H1-B	H1-C	H1-A	H1-B	H1-C
**1**	0.28	0.34	0.42	66	80	100
**2**	0.11	0.36	0.24	31	100	68
**3**	0.17	0.23	0.20	72	100	88
**4**	0.17	0.25	0.24	67	100	94
**5**	0.07	0.06	0.06	100	82	87
**6**	0.08	0.12	0.12	62	100	94
**7**	0.18	0.08	0.11	100	48	64
**8**	-	0.18	0.19	-	99	100

**Table 2:** IC_50_ values for **1**–**8**.

Compound	IC_50_ (μM)
**1**	49.2 ± 0.3
**2**	26.1 ± 0.7
**3**	15.4 ± 0.4
**4**	84.7 ± 0.4
**5**	50.6 ± 0.1
**6**	180.9 ± 5.5
**7**	46.7 ± 0.9
**8**	132.4 ± 1.8

## Supplementary Materials

Supplementary materials can be found at www.mdpi.com/1422-0067/18/6/1293/s1.

## References

[1] Yayon A., Klagsbrun M., Esko J.D., Leder P., Ornitz D.M. (1991). Cell surface, heparin-like molecules are required for binding of basic fibroblast growth factor to its high affinity receptor. Cell.

[2] Eswarakumar V.P., Lax I., Schlessinger J. (2005). Cellular signaling by fibroblast growth factor receptors. Cytokine Growth Factor Rev..

[3] Beenken A., Mohammadi M. (2009). The FGF family: Biology, pathophysiology and therapy. Nat. Rev. Drug Discov..

[4] Schlessinger J., Plotnikov A.N., Ibrahimi O.A., Eliseenkova A.V., Yeh B.K., Yayon A., Linhardt R.J., Mohammadi M. (2000). Crystal structure of a ternary FGF-FGFR-heparin complex reveals a dual role for heparin in FGFR binding and dimerization. Mol. Cell.

[5] Plotnikov A.N., Hubbard S.R., Schlessinger J., Mohammadi M. (2000). Crystal structures of two FGF-FGFR complexes reveal the determinants of ligand-receptor specificity. Cell.

[6] Saxena K., Schieborr U., Anderka O., Duchardt-Ferner E., Elshorst B., Gande S.L., Janzon J., Kudlinzki D., Sreeramulu S., Dreyer M.K. (2010). Influence of heparin mimetics on assembly of the FGF center dot FGFR4 signaling complex. J. Biol. Chem..

[7] DiGabriele A.D., Lax I., Chen D.I., Svahn C.M., Jaye M., Schlessinger J., Hendrickson W.A. (1998). Structure of a heparin-linked biologically active dimer of fibroblast growth factor. Nature.

[8] Canales A., Lozano R., Lopez-Mendez B., Angulo J., Ojeda R., Nieto P.M., Martin-Lomas M., Gimenez-Gallego G., Jimenez-Barbero J. (2006). Solution nmr structure of a human FGF-1 monomer, activated by a hexasaccharide heparin-analogue. FEBS J..

[9] Muñoz-García J.C., García-Jiménez M.J., Carrero P., Canales Á., Jiménez-Barbero J., Martín-Lomas M., Imberty A., de Paz J.L., Angulo J., Lortat-Jacob H. (2014). Importance of the polarity of the glycosaminoglycan chain on the interaction with FGF-1. Glycobiology.

[10] Turnbull J., Powell A., Guimond S. (2001). Heparan sulfate: Decoding a dynamic multifunctional cell regulator. Trends Cell Biol..

[11] Jastrebova N., Vanwildemeersch M., Lindahl U., Spillmann D. (2010). Heparan sulfate domain organization and sulfation modulate FGF-induced cell signaling. J. Biol. Chem..

[12] Guimond S.E., Turnbull J.E. (1999). Fibroblast growth factor receptor signalling is dictated by specific heparan sulphate saccharides. Curr. Biol..

[13] De Paz J.L., Angulo J., Lassaletta J.M., Nieto P.M., Redondo-Horcajo M., Lozano R.M., Gimenez-Gallego G., Martin-Lomas M. (2001). The activation of fibroblast growth factors by heparin: Synthesis, structure, and biological activity of heparin-like oligosaccharides. ChemBioChem.

[14] Munoz-Garcia J.C., Solera C., Carrero P., de Paz J.L., Angulo J., Nieto P.M. (2013). 3D structure of a heparin mimetic analogue of a FGF-1 activator. A nmr and molecular modelling study. Org. Biomol. Chem..

[15] Angulo J., Ojeda R., de Paz J.L., Lucas R., Nieto P.M., Lozano R.M., Redondo-Horcajo M., Gimenez-Gallego G., Martin-Lomas M. (2004). The activation of fibroblast growth factors (FGFs) by glycosaminoglycans: Influence of the sulfation pattern on the biological activity of FGF-1. ChemBioChem.

[16] Roy S., El Hadri A., Richard S., Denis F., Holte K., Duffner J., Yu F., Galcheva-Gargova Z., Capila I., Schultes B. (2014). Synthesis and biological evaluation of a unique heparin mimetic hexasaccharide for structure–activity relationship studies. J. Med. Chem..

[17] Guerrini M., Agulles T., Bisio A., Hricovini M., Lay L., Naggi A., Poletti L., Sturiale L., Torri G., Casu B. (2002). Minimal heparin/heparan sulfate sequences for binding to fibroblast growth factor-1. Biochem. Biophys. Res. Commun..

[18] Hu Y.-P., Zhong Y.-Q., Chen Z.-G., Chen C.-Y., Shi Z., Zulueta M.M.L., Ku C.-C., Lee P.-Y., Wang C.-C., Hung S.-C. (2012). Divergent synthesis of 48 heparan sulfate-based disaccharides and probing the specific sugar–fibroblast growth factor-1 interaction. J. Am. Chem. Soc..

[19] Seeberger P.H. (2008). Automated oligosaccharide synthesis. Chem. Soc. Rev..

[20] Mikhailov D., Linhardt R.J., Mayo K.H. (1997). NMR solution conformation of heparin-derived hexasaccharide. Biochem. J..

[21] Muñoz-García J.C., Corzana F., de Paz J.L., Angulo J., Nieto P.M. (2013). Conformations of the iduronate ring in short heparin fragments described by time averaged distance restrained molecular dynamics. Glycobiology.

[22] Munoz-Garcia J.C., Lopez-Prados J., Angulo J., Diaz-Contreras I., Reichardt N., de Paz J.L., Martin-Lomas M., Nieto P.M. (2012). Effect of the substituents of the neighboring ring in the conformational equilibrium of iduronate in heparin-like trisaccharides. Chem. Eur. J..

[23] Mulloy B., Forster M.J., Jones C., Davies D.B. (1993). NMR and molecular-modeling studies of the solution conformation of heparin. Biochem. J..

[24] Maccarana M., Casu B., Lindahl U. (1993). Minimal sequence in heparin/heparan sulfate required for binding of basic fibroblast growth factor. J. Biol. Chem..

[25] Muñoz-García J.C., Chabrol E., Vivès R.R., Thomas A., de Paz J.L., Rojo J., Imberty A., Fieschi F., Nieto P.M., Angulo J. (2015). Langerin–heparin interaction: Two binding sites for small and large ligands as revealed by a combination of nmr spectroscopy and cross-linking mapping experiments. J. Am. Chem. Soc..

[26] Ojeda R., Angulo J., Nieto P.M., Martin-Lomas M. (2002). The activation of fibroblast growth factors by heparin: Synthesis and structural study of rationally modified heparin-like oligosaccharides. Can. J. Chem.-Rev. Can. Chim..

[27] Lucas R., Angulo J., Nieto P.M., Martin-Lomas M. (2003). Synthesis and structural study of two new heparin-like hexasaccharides. Org. Biomol. Chem..

[28] De Paz J.L., Martin-Lomas M. (2005). Synthesis and biological evaluation of a heparin-like hexasaccharide with the structural motifs for binding to fgf and fgfr. Eur. J. Org. Chem..

[29] Maza S., Kayser M.M., Macchione G., Lopez-Prados J., Angulo J., de Paz J.L., Nieto P.M. (2013). Synthesis of chondroitin/dermatan sulfate-like oligosaccharides and evaluation of their protein affinity by fluorescence polarization. Org. Biomol. Chem..

[30] Mayer M., Meyer B. (1999). Characterization of ligand binding by saturation transfer difference nmr spectroscopy. Angew. Chem. Int. Ed..

[31] Angulo J., Nieto P.M. (2011). STD-NMR: Application to transient interactions between biomolecules-a quantitative approach. Eur. Biophys. J..

[32] Ni F. (1994). Recent developments in transferred noe methods. Prog. Nucl. Magn. Reson. Spectrosc..

[33] Angulo J., Enriquez-Navas P.M., Nieto P.M. (2010). Ligand-receptor binding affinities from saturation transfer difference (STD) NMR spectroscopy: The binding isotherm of std initial growth rates. Chem. Eur. J..

[34] Ornitz D.M., Herr A.B., Nilsson M., Westman J., Svahn C.M., Waksman G. (1995). FGF binding and FGF receptor activation by synthetic heparan-derived di- and trisaccharides. Science.

[35] Jemth P., Kreuger J., Kusche-Gullberg M., Sturiale L., Gimenez-Gallego G., Lindahl U. (2002). Biosynthetic oligosaccharide libraries for identification of protein-binding heparan sulfate motifs—Exploring the structural diversity by screening for fibroblast growth factor (FGF) 1 and FGF2 binding. J. Biol. Chem..

[36] Fielding L. (2007). Nmr methods for the determination of protein-ligand dissociation constants. Prog. Nucl. Magn. Reson. Spectrosc..

[37] (2015). Schrödinger Release 2015–4: Maestro 10.4, G..

[38] Jayalakshmi V., Krishna N.R. (2002). Complete relaxation and conformational exchange matrix (corcema) analysis of intermolecular saturation transfer effects in reversibly forming ligand-receptor complexes. J. Magn. Reson..

[39] Enríquez-Navas P.M., Guzzi C., Muñoz-García J.C., Nieto P.M., Angulo J. (2015). Structures of glycans bound to receptors from saturation transfer difference (STD) NMR spectroscopy: Quantitative analysis by using CORCEMA-ST. Methods Mol. Biol..

[40] Rama Krishna N., Jayalakshmi V. (2006). Complete relaxation and conformational exchange matrix analysis of std-nmr spectra of ligand-receptor complexes. Prog. Nucl. Magn. Reson. Spectrosc..

[41] Mayer M., James T.L. (2004). NMR-based characterization of phenothiazines as a rna binding scaffold. J. Am. Chem. Soc..

[42] Peng J.W., Moore J., Abdul-Manan N. (2004). NMR experiments for lead generation in drug discovery. Prog. Nucl. Magn. Reson. Spectrosc..

[43] Yuan Y., Bleile D.W., Wen X., Sanders D.A.R., Itoh K., Liu H.W., Pinto B.M. (2008). Investigation of binding of UDP-Gal*f* and UDP-[3-F]Gal*f* to udp-galactopyranose mutase by STD-NMR spectroscopy, molecular dynamics, and CORCEMA-ST calculations. J. Am. Chem. Soc..

