# Supplementary material for: Interactions between a Heparin Trisaccharide Library and FGF-1 Analyzed by NMR Methods
Source: Int J Mol Sci. 2017 Jun 17;18(6):1293. doi: 10.3390/ijms18061293 (PMC5486114; doi:10.3390/ijms18061293)
Supplement: Supplementary file 1 [file ijms-18-01293-s001.pdf]

Interactions between a heparin trisaccharide library and FGF-1 analyzed by NMR methods

M. José García-Jiménez,[1] Sergio Gil-Caballero,[1] Ángeles Canales,[2] Jesús Jiménez-Barbero,[3,4,5] José L. de Paz,[1] and Pedro M. Nieto[1,*]

[1] Glycosystems Laboratory, Instituto de Investigaciones Químicas (IIQ), Centro de Investigaciones Científicas Isla de La Cartuja. CSIC and Universidad de Sevilla. Américo Vespucio, 49, 41092 Sevilla, Spain;

[2] Complutense University of Madrid, Fac CC Quim, Dept Quim Organ 1, Avd Complutense S/N, E-28040 Madrid, Spain

[3] CIC bioGUNE, Bizkaia Technology Park, Building 801A, 48170 Derio, Spain.

[4] Basque Foundation for Science, Maria Diaz de Haro 13, 48009, Bilbao, Spain

[5] Department of Organic Chemistry II, Faculty of Science and Technology, University of the Basque Country, 48940, Leioa, Bizkaia, Spain.

* Correspondence: pedro.nieto@iiq.csic.es; Tel.: + 34-954-48-95-68

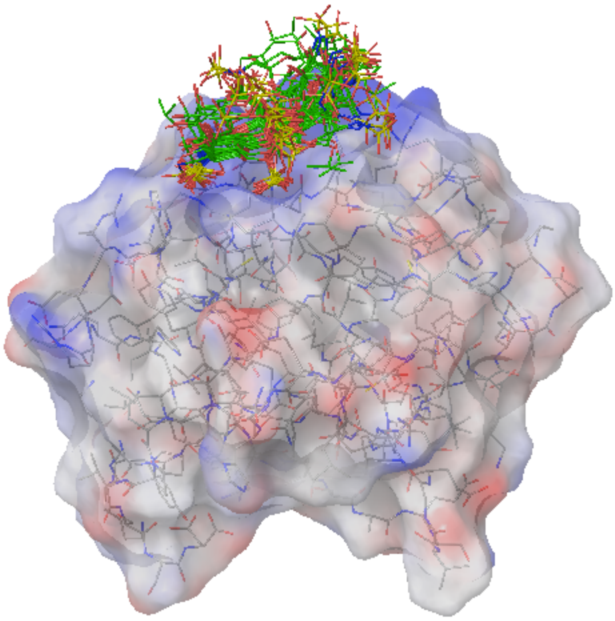

Figure S1. Docking results for **1**. Superimposition of the10 structures with better docking score centered at the canonical binding site.

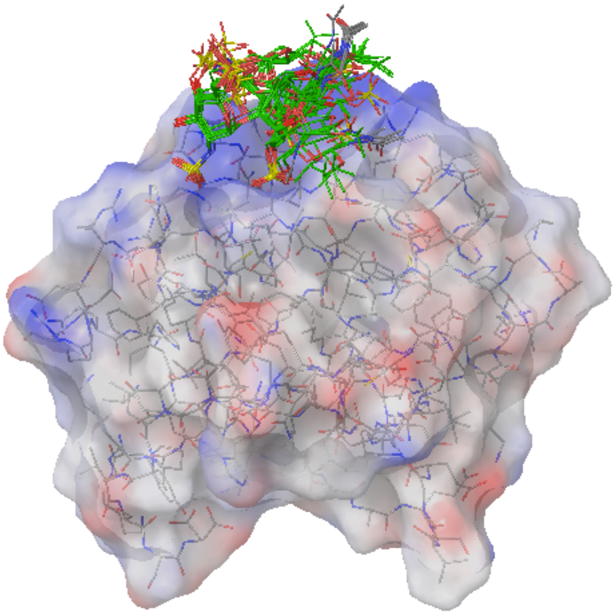

Figure S2. Docking results for **2**. Superimposition of the10 structures with better docking score centered at the canonical binding site.

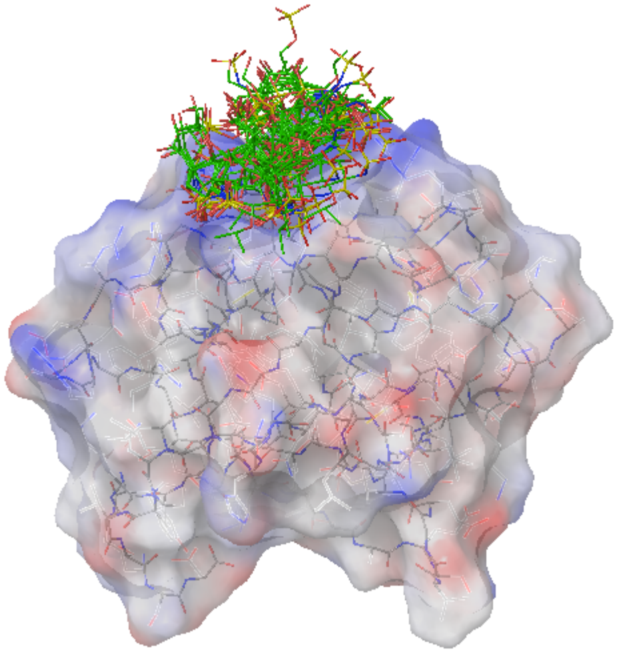

Figure S3. Docking results for **3**. Superimposition of the10 structures with better docking score centered at the canonical binding site.

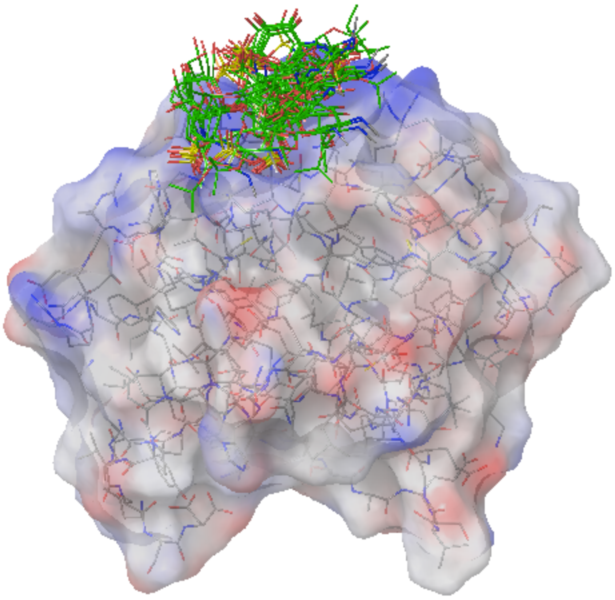

Figure S4. Docking results for **4**. Superimposition of the10 structures with better docking score centered at the canonical binding site.

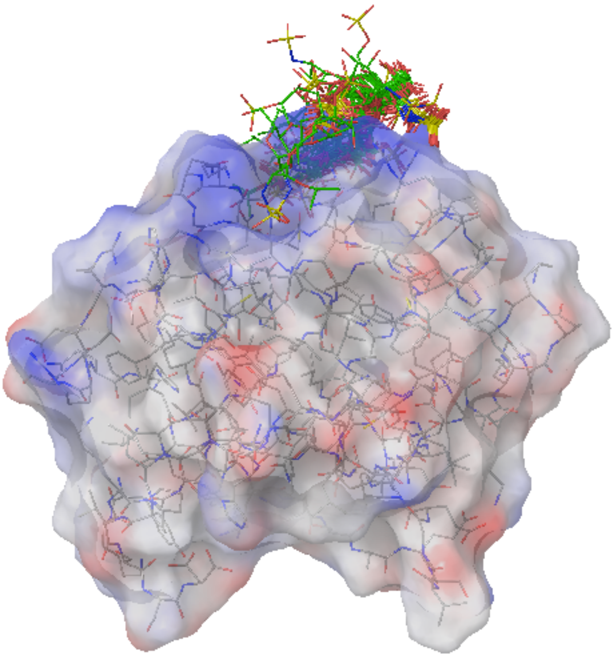

Figure S5. Docking results for **5**. Superimposition of the10 structures with better docking score with the complete protein considered.

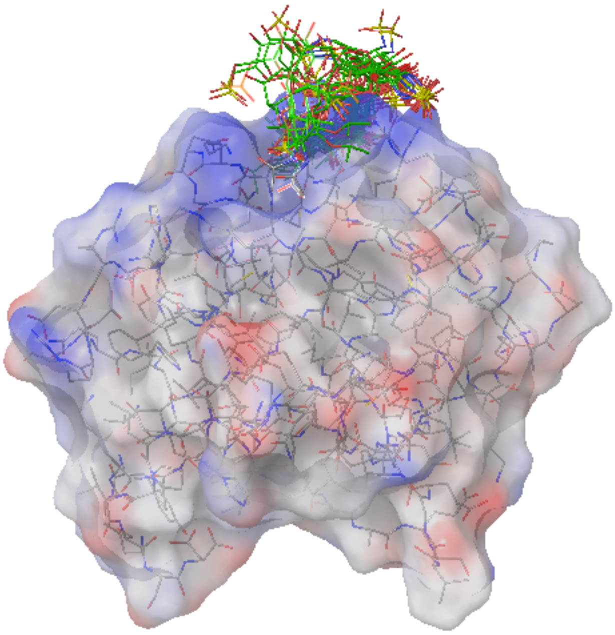

Figure S6. Docking results for **6**. Superimposition of the10 structures with better docking score with the complete protein considered.

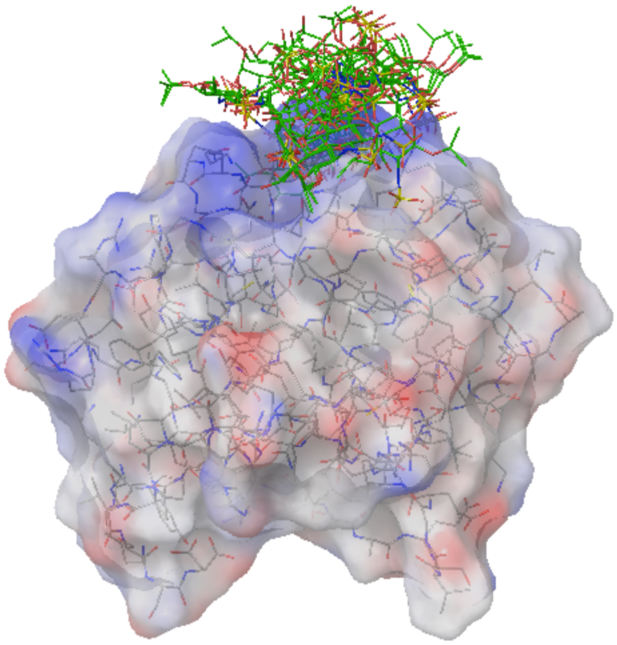

Figure S7. Docking results for **7**. Superimposition of the10 structures with better docking score with the complete protein considered.

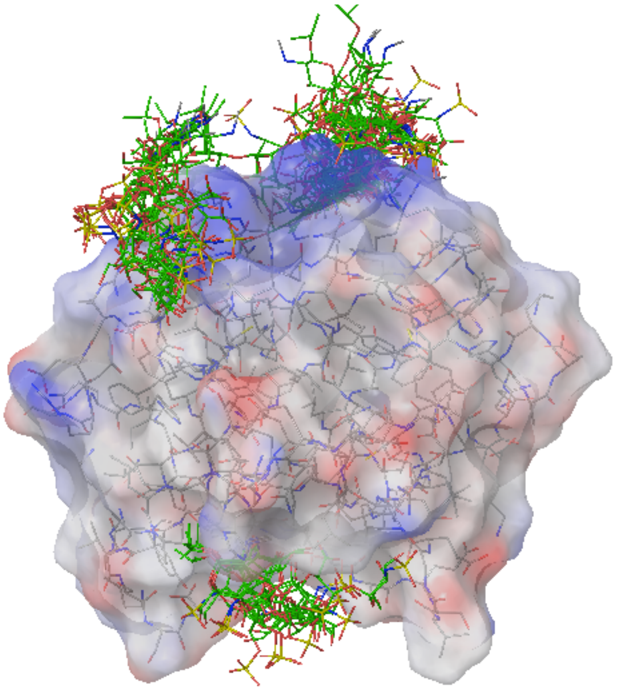

Figure S8. Docking results for **8**. Superimposition of the20 structures with better docking score with the complete protein considered.

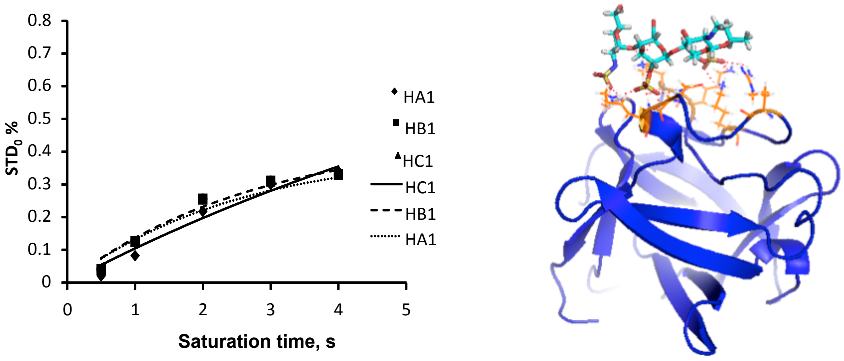

Figure S9. Docking results for compound **3**. Absolute STD growth curve vs irradiation time (dots) and theoretical curves (lines). The structure (left) was obtained from the Docking calculations after energy minimization of the structure with the best docking coefficient.

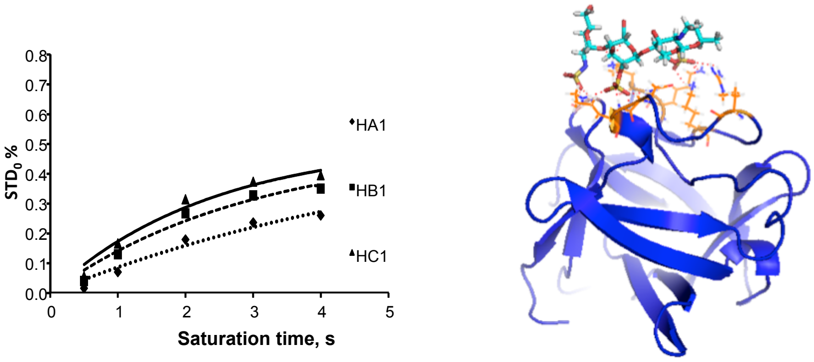

Figure S10. Docking results for compound **4**. Absolute STD growth curve vs irradiation time (dots) and theoretical curves (lines). The structure (left) was obtained from the Docking calculations after energy minimization of the structure with the best docking coefficient.

Calculation of Binding Constants using T_{1sel}.

We have used the T_{1sel} method for the evaluation of the K_D binding. In order to assess the validity of the method we performed a preliminary assay using the known system of the binding of GlcNAc to WGA (Wheat Germen Agglutinin) using a concentration of lectin of 20 uM. We obtained a K_D of 2.1 mM while the reported one is 2.5 mM.
T_{1sel} method is described in the experimental section of the main paper.

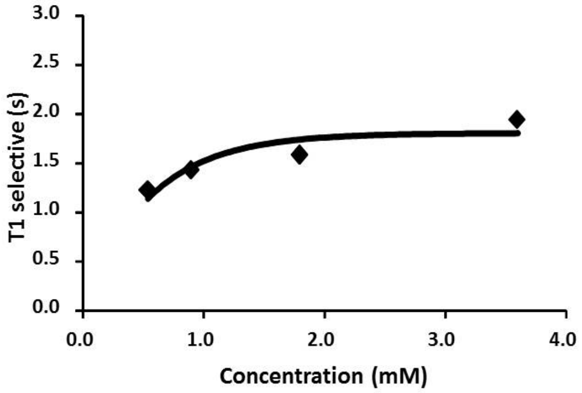

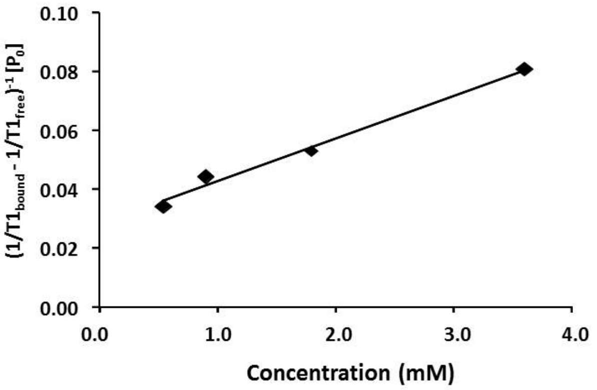

Docking Results

We have performed a virtual screening by docking the ligands (8) using the tools provided by Schrodinger suite (Glide). Glide[1,2] uses a series of hierarchical filters to search for possible locations of the ligand into the binding site. The shape and the properties of the receptor are represented on a grid by different set of fields that are progressively/sequentially applied. The exhaustive generation conformer by modification of the ligand torsions is done in order to generate a collection of possible structures. This are evaluated first into the torsional space and finally in the phase with a strong reduction on the number of hits. This are minimized and further processed.

Virtual screening stages:
1._Generation.- Conformational search of rotatable bonds,
2.- Site-point search. Exhaustive search of possible locations and orientations is performed using a grid of 2A spaced grid covering the active site. The pose is skipped if there is not a good enough mach.
3- Diameter test. If there is too many steric classes with the receptor the orientation is skipped.
4.- Subset test, small rotation about the ligand diameter. It this is good enough the interactions are scored.
5._ Scoring Software owned function of ChemScore.
6.- Energy minimization and annealing
7.- Poses were evaluated according to Schrodinger owned scoring function, including steric-class, buried polar terms, amide twist penalties, hydrophobic enclosure terms, and excluded volume penalties.

Compound **1**

	docking score	glide gscore	glide lipo	glide hbond	glide evdw	glide ecoul	glide erotb	glide esite	glide emodel	glide energy	glide einterna
1	-7.908	-7.908	0	-2.186	-23.172	-59.088	0.072	-0.362	-170.673	-82.26	17.573
2	-7.776	-7.776	-0.007	-2.044	-21.484	-57.423	0.072	-0.301	-163.302	-78.908	11.811
3	-7.749	-7.749	-0.009	-2.012	-19.686	-62.268	0.072	-0.297	-167.913	-81.954	11.335
4	-7.728	-7.728	-0.003	-1.97	-22.427	-55.039	0.072	-0.296	-161.186	-77.466	12.056
5	-7.659	-7.659	-0.004	-1.984	-21.685	-55.53	0.072	-0.401	-164.352	-77.215	14.001
6	-7.648	-7.648	0	-2.04	-21.951	-67.242	0.072	-0.19	-180.947	-89.194	18.87
7	-7.643	-7.643	-0.005	-2.016	-18.549	-59.599	0.072	-0.288	-157.111	-78.148	10.469
8	-7.642	-7.642	0	-1.958	-22.796	-58.77	0.072	-0.187	-162.702	-81.566	21.255
9	-7.628	-7.628	0	-1.966	-20.933	-62.585	0.072	-0.295	-164.421	-83.518	11.31
10	-7.565	-7.565	-0.007	-1.909	-20.103	-56.711	0.072	-0.286	-158.599	-76.814	12.145
11	-7.526	-7.526	-0.006	-1.92	-18.053	-58.358	0.072	-0.284	-159.483	-76.412	11.84
12	-7.516	-7.516	0	-1.938	-19.183	-63.839	0.072	-0.302	-164.362	-83.022	7.897
13	-7.502	-7.502	0	-1.96	-18.551	-65.151	0.072	-0.313	-165.135	-83.701	10.425
14	-7.5	-7.5	-0.001	-2.019	-21.9	-67.237	0.072	-0.174	-176.614	-89.137	30.763
15	-7.464	-7.464	0	-1.919	-17.342	-66.257	0.072	-0.324	-159.33	-83.6	26.802
16	-7.464	-7.464	-0.008	-1.868	-22.07	-55.575	0.072	-0.112	-141.828	-77.645	38.178
17	-7.44	-7.44	0	-1.979	-19.518	-59.241	0.072	-0.304	-153.46	-78.758	16.965
18	-7.383	-7.383	0	-1.482	-23.057	-51.45	0.072	-0.445	-143.17	-74.506	30.216
19	-7.362	-7.362	0	-1.754	-18.217	-55.37	0.072	-0.274	-147.412	-73.588	11.659
20	-7.357	-7.357	0	-2.002	-20.989	-55.806	0.072	-0.302	-160.5	-76.795	13.458
21	-7.35	-7.35	0	-1.899	-18.172	-62.869	0.072	-0.263	-160.548	-81.041	8.285
22	-7.34	-7.34	0	-1.843	-18.992	-61.445	0.072	-0.276	-160.122	-80.437	9.893
23	-7.332	-7.332	0	-1.736	-20.362	-56.211	0.072	-0.301	-156.225	-76.572	7.587
24	-7.317	-7.317	-0.009	-1.684	-19.158	-58.386	0.072	-0.272	-157.639	-77.544	9.751
25	-7.313	-7.313	-0.005	-1.794	-20.285	-57.056	0.072	-0.291	-156.579	-77.34	16.168
26	-7.306	-7.306	-0.002	-1.724	-21.241	-59.889	0.072	-0.302	-160.633	-81.129	15.462
27	-7.294	-7.294	0	-1.803	-18.88	-59.628	0.072	-0.278	-156.176	-78.509	10.194
28	-7.293	-7.293	0	-1.904	-17.467	-59.884	0.072	-0.258	-153.265	-77.351	8.773
29	-7.291	-7.291	0	-1.849	-19.925	-59.788	0.072	-0.268	-153.803	-79.714	14.393
30	-7.29	-7.29	-0.001	-1.554	-21.368	-56.632	0.072	-0.334	-149.211	-78	20.247
31	-7.273	-7.273	-0.007	-1.817	-16.995	-62.63	0.072	-0.362	-146.938	-79.625	26.884
32	-7.27	-7.27	-0.004	-1.59	-21.718	-60.701	0.072	-0.394	-169.847	-82.42	12.26
33	-7.255	-7.255	-0.008	-1.739	-21.31	-58.387	0.072	-0.265	-159.037	-79.698	17.237
34	-7.246	-7.246	0	-1.598	-23.092	-58.916	0.072	-0.264	-157.376	-82.009	24.837
35	-7.232	-7.232	0	-1.9	-17.45	-61.273	0.072	-0.203	-139.327	-78.722	35.282
36	-7.214	-7.214	-0.021	-1.626	-21.607	-54.29	0.072	-0.221	-151.495	-75.897	34.486
37	-7.21	-7.21	0	-1.588	-21.828	-63.151	0.072	-0.281	-160.656	-84.979	16.108
38	-7.208	-7.208	0	-1.687	-20.999	-61.598	0.072	-0.308	-167.227	-82.597	16.265
39	-7.206	-7.206	-0.008	-1.771	-20.303	-55.888	0.072	-0.261	-147.921	-76.191	24.382
40	-7.206	-7.206	0	-1.618	-17.691	-62.069	0.072	-0.181	-151.254	-79.76	12.159
41	-7.203	-7.203	-0.008	-1.69	-19.862	-55.571	0.072	-0.301	-146.175	-75.434	22.468
42	-7.192	-7.192	0	-2.054	-15.948	-64.159	0.072	-0.41	-138.321	-80.106	39.606
43	-7.189	-7.189	0	-1.585	-21.296	-63.768	0.072	-0.288	-158.511	-85.064	20.085
44	-7.186	-7.186	-0.01	-1.743	-16.234	-63.101	0.072	-0.35	-145.267	-79.336	27.719

45	-7.185	-7.185	0	-1.728	-20.166	-56.384	0.072	-0.246	-144.884	-76.55	21.375
46	-7.174	-7.174	-0.001	-1.75	-17.221	-65.76	0.072	-0.194	-153.529	-82.981	24.336
47	-7.162	-7.162	0	-1.919	-19.164	-59.882	0.072	-0.101	-152.277	-79.047	11.388
48	-7.149	-7.149	0	-1.844	-22.384	-62.303	0.072	-0.177	-171.062	-84.687	23.351
49	-7.141	-7.141	-0.006	-1.456	-20.729	-52.299	0.072	-0.26	-151.29	-73.029	9.331
50	-7.136	-7.136	0	-1.639	-15.256	-53.344	0.072	-0.447	-158.768	-68.601	15.393
51	-7.128	-7.128	-0.007	-1.484	-19.573	-52.523	0.072	-0.269	-150.857	-72.097	8.033
52	-7.123	-7.123	0	-1.594	-20.053	-61.519	0.072	-0.312	-157.126	-81.572	19.008
53	-7.121	-7.121	0	-1.828	-20.595	-61.333	0.072	-0.139	-154.712	-81.928	12.098
54	-7.106	-7.106	-0.038	-1.636	-19.674	-56.758	0.072	-0.272	-146.46	-76.432	20.165
55	-7.085	-7.085	0	-1.732	-15.313	-65.268	0.072	-0.337	-152.346	-80.581	25.659
56	-7.083	-7.083	0	-1.568	-19.028	-56.915	0.072	-0.31	-153.539	-75.942	12.307
57	-7.074	-7.074	0	-1.703	-17.909	-61.669	0.072	-0.271	-152.133	-79.579	12.63
58	-7.065	-7.065	0	-1.861	-16.664	-52.829	0.072	-0.331	-140.238	-69.493	12.618
59	-7.058	-7.058	-0.017	-1.785	-18.096	-61.251	0.072	-0.215	-150.781	-79.347	21.508
60	-7.047	-7.047	0	-1.904	-15.792	-55.558	0.072	-0.102	-144.133	-71.351	11.446
61	-7.047	-7.047	0	-1.837	-18.607	-58.407	0.072	-0.106	-149.024	-77.014	13.104
62	-7.042	-7.042	0	-1.761	-16.935	-57.608	0.072	-0.322	-144.113	-74.543	18.33
63	-7.042	-7.042	0	-1.9	-17.433	-63.645	0.072	-0.103	-149.139	-81.078	16.259
64	-7.035	-7.035	-0.01	-1.591	-18.494	-57.138	0.072	-0.276	-151.668	-75.632	11.31
65	-7.031	-7.031	0	-1.869	-17.808	-62.136	0.072	-0.103	-155.257	-79.944	6.582
66	-7.022	-7.022	-0.004	-1.477	-19.673	-58.63	0.072	-0.342	-166.972	-78.303	15.266
67	-7.021	-7.021	0	-1.802	-17.606	-57.165	0.072	-0.083	-162.489	-74.771	10.367
68	-7.019	-7.019	-0.004	-1.542	-19.361	-55.191	0.072	-0.284	-152.46	-74.552	10.162
69	-7.018	-7.018	0	-1.604	-18.696	-54.355	0.072	-0.251	-147.454	-73.051	10.933
70	-7.016	-7.016	0	-1.757	-20.308	-58.642	0.072	-0.11	-149.416	-78.95	10.879
71	-7.016	-7.016	0	-1.54	-18.52	-57.221	0.072	-0.291	-154.078	-75.741	15.25
72	-7.011	-7.011	0	-1.709	-19.265	-57.407	0.072	-0.139	-142.583	-76.672	23.871
73	-7.008	-7.008	0	-1.559	-15.661	-50.116	0.072	-0.36	-135.425	-65.778	12.348
74	-7.003	-7.003	-0.019	-1.855	-18.85	-62.975	0.072	-0.122	-159.123	-81.825	17.003
75	-6.988	-6.988	-0.003	-1.575	-19.228	-54.376	0.072	-0.286	-145.277	-73.604	10.609
76	-6.977	-6.977	-0.007	-1.526	-17.659	-59.609	0.072	-0.276	-150.961	-77.268	14.661
77	-6.969	-6.969	0	-1.776	-18.742	-61.486	0.072	-0.119	-151.515	-80.227	11.026
78	-6.969	-6.969	0	-1.552	-18.587	-51.817	0.072	-0.267	-146.946	-70.404	14.285
79	-6.963	-6.963	0	-1.76	-18.628	-60.227	0.072	-0.091	-151.965	-78.855	7.237
80	-6.962	-6.962	0	-1.565	-17.847	-56.86	0.072	-0.25	-143.06	-74.707	21.67
81	-6.96	-6.96	0	-1.996	-14.718	-65.217	0.072	-0.132	-150.795	-79.935	13.901
82	-6.958	-6.958	0	-1.612	-19.462	-52.73	0.072	-0.276	-147.858	-72.192	9.178
83	-6.954	-6.954	0	-1.6	-20.64	-59.518	0.072	-0.279	-154.516	-80.159	13.253
84	-6.952	-6.952	0	-1.775	-20.779	-57.569	0.072	-0.117	-155.083	-78.348	16.891
85	-6.951	-6.951	0	-1.487	-20.329	-56.091	0.072	-0.343	-151.038	-76.42	15.146
86	-6.949	-6.949	0	-1.704	-17.766	-50.961	0.072	-0.318	-140.879	-68.728	12.125
87	-6.945	-6.945	0	-1.736	-18.915	-58.147	0.072	-0.099	-149.782	-77.061	14.716
88	-6.938	-6.938	0	-1.617	-16.852	-63.083	0.072	-0.185	-148.968	-79.935	11.648
89	-6.938	-6.938	0	-1.689	-17.476	-52.355	0.072	-0.307	-138.288	-69.831	16.064
90	-6.933	-6.933	0	-1.906	-15.679	-58.402	0.072	-0.093	-141.051	-74.081	15.748
91	-6.932	-6.932	0	-1.714	-15.389	-50.451	0.072	-0.376	-136.529	-65.84	17.191
92	-6.931	-6.931	0	-1.849	-16.946	-61.407	0.072	-0.107	-150.773	-78.352	9.695
93	-6.927	-6.927	0	-1.93	-15.319	-63.763	0.072	-0.13	-148.968	-79.083	15.11
94	-6.926	-6.926	0	-1.377	-22.449	-52.038	0.072	-0.317	-151.682	-74.486	23.881

95	-6.921	-6.921	0	-1.482	-19.616	-52.145	0.072	-0.205	-149	-71.761	13.229
96	-6.92	-6.92	0	-1.558	-19.747	-49.899	0.072	-0.359	-146.803	-69.646	11.886
97	-6.918	-6.918	-0.024	-1.557	-17.634	-54.041	0.072	-0.282	-137.333	-71.675	17.213
98	-6.915	-6.915	0	-1.775	-15.66	-56.276	0.072	-0.09	-145.656	-71.936	6.625
99	-6.915	-6.915	0	-1.572	-20.309	-48.828	0.072	-0.281	-136.882	-69.137	13.386
100	-6.908	-6.908	0	-1.748	-17.891	-61.216	0.072	-0.165	-145.39	-79.107	22.921
101	-6.907	-6.907	0	-1.662	-18.577	-54.084	0.072	-0.122	-141.878	-72.661	15.687
102	-6.904	-6.904	0	-1.558	-19.835	-46.549	0.072	-0.307	-131.162	-66.384	18.687
103	-6.898	-6.898	0	-1.875	-14.976	-63.975	0.072	-0.117	-151.275	-78.951	9.156
104	-6.894	-6.894	0	-1.695	-16.733	-51.679	0.072	-0.317	-140.454	-68.411	11.084
105	-6.893	-6.893	0	-1.472	-17.913	-56.11	0.072	-0.218	-145.761	-74.022	8.141
106	-6.891	-6.891	0	-1.606	-18.505	-50.739	0.072	-0.324	-132.933	-69.244	21.695
107	-6.891	-6.891	0	-1.739	-18.558	-49.726	0.072	-0.181	-133.557	-68.284	17.815
108	-6.89	-6.89	0	-1.597	-17.707	-49.717	0.072	-0.48	-135.487	-67.424	12.631
109	-6.887	-6.887	0	-1.512	-20.351	-50.206	0.072	-0.305	-141.015	-70.557	12.019
110	-6.878	-6.878	0	-1.55	-16.614	-55.528	0.072	-0.141	-139.851	-72.142	13.817
111	-6.876	-6.876	0	-1.833	-15.067	-56.771	0.072	-0.356	-148.517	-71.838	13.266
112	-6.863	-6.863	0	-1.745	-17.352	-61.796	0.072	-0.225	-150.11	-79.148	10.535
113	-6.862	-6.862	0	-1.834	-16.107	-57.031	0.072	-0.083	-139.887	-73.138	14.652
114	-6.86	-6.86	-0.022	-1.651	-20.61	-54.495	0.072	-0.228	-147.928	-75.105	10.506
115	-6.858	-6.858	-0.048	-1.433	-18.095	-49.646	0.072	-0.3	-131.975	-67.742	19.893
116	-6.857	-6.857	0	-1.758	-17.243	-57.643	0.072	-0.059	-137.847	-74.886	22.33
117	-6.85	-6.85	0	-1.631	-16.666	-53.782	0.072	-0.294	-137.004	-70.449	17.945
118	-6.848	-6.848	-0.023	-1.294	-15.983	-56.462	0.072	-0.443	-139.42	-72.445	20.449
119	-6.842	-6.842	-0.003	-1.512	-21.513	-55.154	0.072	-0.205	-150.321	-76.667	18.598
120	-6.835	-6.835	0	-1.719	-17.271	-61.433	0.072	-0.293	-149.828	-78.704	18.098
121	-6.832	-6.832	0	-1.416	-22.434	-50.944	0.072	-0.197	-137.569	-73.379	22.196
122	-6.83	-6.83	-0.003	-1.699	-19.298	-54.801	0.072	-0.234	-156.229	-74.099	16.464
123	-6.826	-6.826	0	-1.461	-19.408	-50.654	0.072	-0.081	-154.55	-70.061	11.659
124	-6.822	-6.822	0	-1.399	-16.412	-52.143	0.072	-0.315	-132.906	-68.555	25.008
125	-6.822	-6.822	0	-1.767	-18.173	-56.845	0.072	-0.105	-142.877	-75.019	21.869
126	-6.821	-6.821	0	-1.74	-17.297	-59.437	0.072	-0.289	-153.581	-76.734	13.997
127	-6.812	-6.812	0	-1.636	-17.265	-52.85	0.072	-0.254	-135.854	-70.115	19.853
128	-6.803	-6.803	0	-1.299	-18.263	-58.754	0.072	-0.356	-140.01	-77.017	41.258
129	-6.803	-6.803	0	-1.569	-19.396	-50.105	0.072	-0.248	-145.258	-69.501	11.864
130	-6.802	-6.802	0	-1.632	-20.107	-58.254	0.072	-0.236	-147.294	-78.361	17.443
131	-6.791	-6.791	0	-1.629	-17.604	-51.554	0.072	-0.076	-141.016	-69.159	6.651
132	-6.791	-6.791	0	-1.597	-18.402	-57.368	0.072	-0.176	-143.339	-75.77	16.739
133	-6.79	-6.79	0	-1.274	-21.72	-49.562	0.072	-0.322	-155.018	-71.283	18.783
134	-6.789	-6.789	0	-1.466	-15.996	-59.06	0.072	-0.37	-147.271	-75.056	21.938
135	-6.788	-6.788	0	-1.465	-17.675	-60.305	0.072	-0.28	-146.396	-77.98	13.974
136	-6.776	-6.776	-0.018	-1.563	-22.183	-55.146	0.072	-0.158	-150.985	-77.329	8.946
137	-6.771	-6.771	0	-1.575	-18.135	-53.832	0.072	-0.102	-142.36	-71.967	9.635
138	-6.77	-6.77	0	-1.525	-18.974	-54.385	0.072	-0.075	-137.551	-73.359	16.564
139	-6.766	-6.766	-0.006	-1.525	-22.025	-55.793	0.072	-0.164	-143.837	-77.818	16.976
140	-6.763	-6.763	-0.02	-1.595	-20.579	-57.931	0.072	-0.121	-144.062	-78.51	22.383
141	-6.76	-6.76	0	-1.346	-20.565	-52.965	0.072	-0.397	-151.968	-73.53	17.122
142	-6.751	-6.751	-0.021	-1.574	-20.829	-57.597	0.072	-0.185	-148.035	-78.426	9.45
143	-6.748	-6.748	0	-1.546	-18.152	-58.785	0.072	-0.197	-136.514	-76.937	18.358
144	-6.746	-6.746	0	-1.512	-18.985	-47.59	0.072	-0.357	-133.977	-66.575	12.343

145	-6.746	-6.746	0	-1.573	-18.592	-46.234	0.072	-0.173	-134.685	-64.826	12.261
146	-6.739	-6.739	-0.007	-1.621	-19.49	-57.198	0.072	-0.2	-139.262	-76.688	26.796
147	-6.734	-6.734	0	-1.494	-18.666	-59.644	0.072	-0.214	-150.277	-78.309	16.715
148	-6.731	-6.731	-0.009	-1.434	-16.938	-50.315	0.072	-0.279	-134.057	-67.253	5.497
149	-6.73	-6.73	0	-1.68	-17.163	-56.73	0.072	-0.263	-142.42	-73.894	11.343
150	-6.729	-6.729	0	-1.279	-15.666	-51.993	0.072	-0.404	-132.657	-67.659	15.496
151	-6.725	-6.725	0	-1.539	-19.735	-55.945	0.072	-0.155	-147.636	-75.68	7.714
152	-6.721	-6.721	0	-1.457	-18.259	-52.893	0.072	-0.304	-138.858	-71.152	18.328
153	-6.717	-6.717	0	-1.514	-16.658	-53.996	0.072	-0.076	-135.197	-70.654	15.523
154	-6.713	-6.713	0	-1.559	-20.207	-47.582	0.072	-0.178	-128.401	-67.789	25.805
155	-6.713	-6.713	-0.02	-1.8	-16.285	-57.6	0.072	-0.14	-144.906	-73.885	13.017
156	-6.712	-6.712	0	-1.583	-20.485	-55.908	0.072	-0.122	-147.782	-76.393	10.47
157	-6.705	-6.705	0	-1.5	-17.638	-46.78	0.072	-0.29	-125.047	-64.417	15.372
158	-6.703	-6.703	-0.014	-1.544	-19.951	-55.662	0.072	-0.22	-133.916	-75.613	31.003
159	-6.702	-6.702	0	-1.569	-14.166	-49.701	0.072	-0.246	-124.618	-63.867	23.12
160	-6.702	-6.702	0	-1.465	-18.997	-52.444	0.072	-0.252	-140.103	-71.441	12.571
161	-6.701	-6.701	0	-1.41	-19.558	-48.797	0.072	-0.292	-137.224	-68.355	9.899
162	-6.701	-6.701	0	-1.558	-15.978	-54.167	0.072	-0.044	-138.015	-70.144	12.626
163	-6.701	-6.701	-0.013	-1.559	-16.812	-52.285	0.072	-0.276	-131.317	-69.098	20.457
164	-6.7	-6.7	0	-1.459	-21.253	-55.182	0.072	-0.151	-145.342	-76.435	13.63
165	-6.69	-6.69	0	-1.311	-20.913	-59.738	0.072	-0.292	-149.727	-80.651	12.656
166	-6.681	-6.681	0	-1.479	-19.666	-58.56	0.072	-0.291	-145.244	-78.226	20.286
167	-6.679	-6.679	-0.005	-1.663	-17.401	-58.037	0.072	-0.212	-142.645	-75.438	19.599
168	-6.675	-6.675	0	-1.428	-18.871	-49.689	0.072	-0.313	-132.133	-68.56	19.098
169	-6.673	-6.673	0	-1.605	-18.858	-51.23	0.072	-0.19	-133.504	-70.088	17.517
170	-6.671	-6.671	0	-1.528	-16.675	-55.445	0.072	-0.289	-141.053	-72.12	14.742
171	-6.669	-6.669	0	-1.325	-18.917	-52.085	0.072	-0.294	-143.963	-71.002	7.916
172	-6.664	-6.664	0	-1.538	-20.152	-58.104	0.072	-0.129	-149.678	-78.256	9.325
173	-6.658	-6.658	-0.016	-1.156	-21.748	-47.271	0.072	-0.116	-128.295	-69.018	17.13
174	-6.648	-6.648	0	-1.536	-17.094	-56.948	0.072	-0.292	-144.07	-74.042	11.263
175	-6.645	-6.645	0	-1.482	-16.055	-56.22	0.072	-0.254	-137.137	-72.275	17.907
176	-6.638	-6.638	0	-1.375	-20.915	-53.039	0.072	-0.144	-135.143	-73.954	19.745
177	-6.638	-6.638	0	-1.272	-20.05	-49.816	0.072	-0.277	-142.222	-69.866	8.406
178	-6.629	-6.629	0	-1.401	-21.065	-51.672	0.072	-0.218	-137.281	-72.737	16.91
179	-6.621	-6.621	0	-1.194	-20.834	-56.065	0.072	-0.17	-142.719	-76.899	14.346
180	-6.613	-6.613	-0.001	-1.344	-18.836	-48.631	0.072	-0.279	-139.329	-67.466	8.832
181	-6.613	-6.613	0	-1.409	-17.632	-48.03	0.072	-0.154	-130.196	-65.662	13.919
182	-6.611	-6.611	0	-1.533	-18.295	-55.36	0.072	-0.235	-142.286	-73.655	13.223
183	-6.607	-6.607	0	-1.367	-19.367	-49.85	0.072	-0.342	-136.583	-69.217	10.788
184	-6.598	-6.598	-0.001	-1.376	-18.658	-49.81	0.072	-0.298	-134.357	-68.469	9.895
185	-6.589	-6.589	0	-1.42	-17.688	-57.982	0.072	-0.194	-139.154	-75.67	21.333
186	-6.584	-6.584	0	-1.548	-16.361	-59.802	0.072	-0.179	-143.343	-76.163	14.057
187	-6.582	-6.582	0	-1.265	-17.268	-50.651	0.072	-0.168	-136.289	-67.919	11.544
188	-6.575	-6.575	0	-1.404	-15.871	-50.897	0.072	-0.341	-131.532	-66.768	16.058
189	-6.575	-6.575	0	-1.454	-13.993	-57.135	0.072	-0.348	-143.915	-71.128	8.651
190	-6.574	-6.574	0	-1.551	-17.659	-49.907	0.072	-0.212	-135.334	-67.566	13.063
191	-6.573	-6.573	0	-1.331	-19.983	-45.864	0.072	-0.218	-136.323	-65.847	15.12
192	-6.572	-6.572	0	-1.643	-16.721	-56.719	0.072	-0.105	-136.744	-73.44	15.288
193	-6.572	-6.572	0	-1.357	-19.677	-50.972	0.072	-0.137	-141.853	-70.649	5.746
194	-6.557	-6.557	0	-1.431	-15.543	-48.234	0.072	-0.315	-129.754	-63.776	13.65

195	-6.553	-6.553	0	-1.528	-16.506	-53.324	0.072	-0.262	-141.42	-69.83	10.548
196	-6.549	-6.549	0	-1.569	-13.711	-57.693	0.072	-0.307	-135.184	-71.404	26.069
197	-6.547	-6.547	0	-1.147	-17.414	-45.33	0.072	-0.234	-121.249	-62.745	17.089
198	-6.547	-6.547	-0.003	-1.473	-16.868	-51.001	0.072	-0.28	-132.138	-67.869	12.023
199	-6.546	-6.546	0	-1.133	-18.902	-52.443	0.072	-0.257	-130.509	-71.345	26.819
200	-6.542	-6.542	0	-1.572	-15.333	-50.232	0.072	-0.074	-131.964	-65.566	9.904
201	-6.537	-6.537	-0.059	-1.378	-19.191	-51.265	0.072	-0.211	-135.694	-70.457	24.749
202	-6.536	-6.536	0	-1.283	-15.899	-44.001	0.072	-0.188	-123.431	-59.9	14.278
203	-6.534	-6.534	0	-1.638	-14.368	-56.164	0.072	-0.244	-135.123	-70.531	18.12
204	-6.534	-6.534	0	-1.543	-17.887	-57.875	0.072	-0.169	-142.191	-75.762	12.023
205	-6.53	-6.53	0	-1.455	-15.25	-54.788	0.072	-0.171	-131.249	-70.038	20.647
206	-6.525	-6.525	0	-1.396	-15.861	-50.129	0.072	-0.408	-132.03	-65.99	19.886
207	-6.515	-6.515	0	-1.434	-18.582	-49.53	0.072	-0.224	-137.539	-68.112	19.132
208	-6.513	-6.513	0	-1.405	-18.155	-48.872	0.072	-0.272	-134.022	-67.027	10.427
209	-6.509	-6.509	0	-1.39	-18.388	-52.333	0.072	-0.271	-131.815	-70.721	21.218
210	-6.507	-6.507	0	-1.444	-17.804	-53.973	0.072	-0.245	-136.743	-71.777	20.132
211	-6.501	-6.501	0	-1.415	-17.407	-49.968	0.072	-0.287	-138.526	-67.375	15.34
212	-6.499	-6.499	-0.01	-1.283	-20.511	-50.445	0.072	-0.099	-134.685	-70.957	17.556
213	-6.498	-6.498	0	-1.384	-16.418	-55.332	0.072	-0.209	-125.074	-71.75	26.445
214	-6.498	-6.498	0	-1.344	-18.997	-47.026	0.072	-0.276	-129.41	-66.024	13.441
215	-6.496	-6.496	0	-1.443	-15.948	-51.205	0.072	-0.327	-132.505	-67.153	24.254
216	-6.483	-6.483	0	-1.29	-17.072	-48.339	0.072	-0.303	-134.884	-65.411	9.353
217	-6.48	-6.48	0	-1.318	-17.83	-56.765	0.072	-0.198	-140.538	-74.595	16.083
218	-6.476	-6.476	0	-1.38	-17.513	-50.382	0.072	-0.145	-130.497	-67.895	16.619
219	-6.459	-6.459	0	-1.252	-17.257	-51.865	0.072	-0.405	-131.599	-69.122	22.069
220	-6.456	-6.456	0	-1.353	-19.51	-52.93	0.072	-0.159	-139.703	-72.439	11.388
221	-6.451	-6.451	0	-1.381	-16.921	-53.436	0.072	-0.27	-137.108	-70.357	10.78
222	-6.45	-6.45	0	-1.216	-20.757	-48.814	0.072	-0.269	-136.345	-69.57	11.862
223	-6.448	-6.448	0	-1.077	-21.423	-44.629	0.072	-0.372	-134.189	-66.053	6.493
224	-6.448	-6.448	-0.022	-1.216	-21.541	-51.974	0.072	-0.205	-134.378	-73.515	16.317
225	-6.447	-6.447	0	-1.372	-18.353	-51.722	0.072	-0.09	-133.824	-70.074	13.754
226	-6.443	-6.443	0	-1.359	-17.654	-47.159	0.072	-0.273	-121.889	-64.813	15.104
227	-6.442	-6.442	-0.011	-1.345	-20.077	-47.147	0.072	-0.152	-133.72	-67.224	8.979
228	-6.439	-6.439	0	-1.373	-19.143	-50.855	0.072	-0.181	-135.414	-69.998	10.796
229	-6.437	-6.437	0	-1.448	-17.366	-51.902	0.072	-0.114	-134.604	-69.267	8.238
230	-6.435	-6.435	0	-1.308	-18.271	-59.075	0.072	-0.285	-148.573	-77.346	11.048
231	-6.429	-6.429	0	-1.18	-18.674	-51.474	0.072	-0.189	-134.686	-70.148	9.99
232	-6.428	-6.428	0	-1.242	-19.521	-52.465	0.072	-0.242	-140.427	-71.986	8.397
233	-6.427	-6.427	0	-1.331	-19.871	-44.625	0.072	-0.163	-126.926	-64.496	15.908
234	-6.425	-6.425	0	-1.227	-19.386	-51.965	0.072	-0.238	-130.812	-71.351	19.722
235	-6.424	-6.424	0	-1.261	-15.074	-49.85	0.072	-0.233	-129.373	-64.924	10.021
236	-6.415	-6.415	0	-1.364	-17.995	-54.596	0.072	-0.222	-141.874	-72.591	13.741
237	-6.413	-6.413	0	-1.231	-17.279	-48.782	0.072	-0.136	-132.635	-66.061	5.531
238	-6.402	-6.402	-0.006	-1.172	-23.089	-48.503	0.072	-0.135	-135.73	-71.593	17.355
239	-6.401	-6.401	0	-1.442	-15.98	-50.055	0.072	-0.096	-135.065	-66.035	9.541
240	-6.4	-6.4	0	-1.345	-16.592	-55.382	0.072	-0.297	-135.375	-71.974	20.771
241	-6.391	-6.391	-0.013	-1.275	-19.884	-53.304	0.072	-0.181	-137.339	-73.188	14.092
242	-6.382	-6.382	0	-1.082	-22.188	-50.063	0.072	-0.087	-131.192	-72.252	19.153
243	-6.382	-6.382	0	-1.364	-17.143	-52.403	0.072	-0.233	-127.391	-69.546	22.098
244	-6.382	-6.382	0	-1.378	-15.358	-56.159	0.072	-0.308	-129.068	-71.517	13.308

245	-6.377	-6.377	0	-1.285	-18.242	-45.912	0.072	-0.223	-129.507	-64.154	9.56
246	-6.366	-6.366	0	-1.367	-16.782	-47.85	0.072	-0.231	-125.467	-64.632	17.186
247	-6.359	-6.359	-0.001	-1.214	-20.726	-47.924	0.072	-0.09	-129.704	-68.65	7.468
248	-6.354	-6.354	0	-1.247	-15.905	-47.642	0.072	-0.211	-121.217	-63.547	12.518
249	-6.354	-6.354	0	-1.141	-19.645	-52.99	0.072	-0.044	-138.383	-72.635	7.705
250	-6.353	-6.353	0	-0.955	-19.378	-44.877	0.072	-0.201	-120.934	-64.256	14.097
251	-6.353	-6.353	0	-1.203	-17.403	-44.109	0.072	-0.239	-116.062	-61.512	18.726
252	-6.351	-6.351	0	-1.463	-16.52	-51.086	0.072	-0.135	-125.596	-67.606	13.449
253	-6.348	-6.348	0	-1.298	-16.798	-53.586	0.072	-0.281	-148.783	-70.384	11.175
254	-6.344	-6.344	0	-1.224	-17.723	-54.693	0.072	-0.197	-130.672	-72.416	20.076
255	-6.34	-6.34	0	-1.31	-14.875	-51.976	0.072	-0.331	-128.2	-66.851	22.327
256	-6.334	-6.334	0	-1.165	-16.99	-52.846	0.072	-0.22	-135.157	-69.836	8.682
257	-6.33	-6.33	0	-1.311	-17.681	-53.146	0.072	-0.121	-131.706	-70.827	19.474
258	-6.327	-6.327	0	-1.283	-17.846	-42.932	0.072	-0.221	-114.084	-60.778	22.975
259	-6.327	-6.327	0	-1.267	-18.592	-49.454	0.072	-0.182	-132.278	-68.045	13.037
260	-6.321	-6.321	0	-1.064	-16.277	-59.501	0.072	-0.232	-139.962	-75.778	13.908
261	-6.316	-6.316	0	-1.358	-15.321	-51.052	0.072	-0.264	-124.109	-66.373	15.064
262	-6.315	-6.315	-0.007	-0.98	-23.161	-48.272	0.072	-0.145	-135.205	-71.433	16.118
263	-6.311	-6.311	0	-1.131	-16.222	-49.254	0.072	-0.398	-128.738	-65.477	12.481
264	-6.307	-6.307	0	-1.382	-14.689	-52.763	0.072	-0.262	-122.549	-67.452	16.34
265	-6.306	-6.306	0	-1.431	-13.933	-43.405	0.072	-0.198	-113.847	-57.337	13.494
266	-6.3	-6.3	0	-1.366	-15.379	-51.485	0.072	-0.236	-138.07	-66.864	10.354
267	-6.296	-6.296	0	-1.261	-12.725	-52.401	0.072	-0.25	-130.524	-65.126	12.442
268	-6.294	-6.294	0	-1.081	-16.158	-48.643	0.072	-0.204	-134.025	-64.802	8.143
269	-6.274	-6.274	0	-1.3	-16.224	-51.301	0.072	-0.235	-131.145	-67.525	23.434
270	-6.274	-6.274	0	-1.265	-18.445	-47.839	0.072	-0.134	-123.729	-66.284	23.296
271	-6.271	-6.271	-0.006	-1.309	-16.244	-43.272	0.072	-0.203	-117.083	-59.516	11.04
272	-6.263	-6.263	0	-1.155	-14.664	-56.4	0.072	-0.207	-130.206	-71.063	26.144
273	-6.251	-6.251	0	-1.011	-20.012	-44.755	0.072	-0.262	-130.228	-64.767	10.369
274	-6.237	-6.237	0	-1.235	-15.941	-52.673	0.072	-0.277	-132.798	-68.614	20.422
275	-6.231	-6.231	0	-1.197	-18	-56.334	0.072	-0.206	-142.153	-74.334	12.308
276	-6.229	-6.229	0	-1.14	-13.36	-52.74	0.072	-0.356	-135.434	-66.101	7.451
277	-6.225	-6.225	0	-1.147	-18.575	-45.317	0.072	-0.197	-124.433	-63.892	15.252
278	-6.215	-6.215	0	-1.193	-16.525	-51.691	0.072	-0.267	-134.735	-68.216	10.282
279	-6.208	-6.208	0	-1.155	-17.73	-58.203	0.072	-0.225	-135.557	-75.933	14.997
280	-6.191	-6.191	0	-1.137	-18.235	-53.461	0.072	-0.214	-139.634	-71.696	11.118
281	-6.187	-6.187	0	-1.255	-13.883	-53.939	0.072	-0.31	-127.841	-67.822	20.708
282	-6.186	-6.186	-0.052	-1.189	-15.902	-46.507	0.072	-0.222	-120.429	-62.409	8.422
283	-6.15	-6.15	0	-1.095	-17.587	-51.849	0.072	-0.141	-132.988	-69.436	11.452
284	-6.149	-6.149	0	-1.108	-16.65	-55.625	0.072	-0.183	-130.363	-72.275	18.543
285	-6.146	-6.146	-0.004	-1.039	-18.025	-43.459	0.072	-0.236	-116.905	-61.484	22.044
286	-6.145	-6.145	0	-1.264	-14.928	-42.682	0.072	-0.206	-112.056	-57.611	16.977
287	-6.145	-6.145	0	-1.072	-15.185	-41.983	0.072	-0.385	-119.774	-57.168	8.815
288	-6.141	-6.141	0	-1.311	-14.968	-52.316	0.072	-0.1	-129.116	-67.284	10.836
289	-6.138	-6.138	0	-1.197	-17.411	-50.554	0.072	-0.142	-132.852	-67.966	8.509
290	-6.129	-6.129	0	-1.072	-18.728	-48.871	0.072	-0.189	-126.604	-67.599	13.211
291	-6.123	-6.123	0	-1.196	-14.342	-53.36	0.072	-0.282	-124.113	-67.701	19.626
292	-6.096	-6.096	0	-1.059	-16.073	-58.585	0.072	-0.258	-124.123	-74.659	36.223
293	-6.079	-6.079	0	-0.886	-20.524	-50.376	0.072	-0.138	-129.615	-70.9	12.717
294	-6.072	-6.072	0	-1.197	-12.706	-49.104	0.072	-0.311	-130.366	-61.81	11.681

295	-6.041	-6.041	0	-0.984	-14.339	-54.566	0.072	-0.342	-129.611	-68.906	12.534
296	-6.035	-6.035	0	-0.835	-19.044	-43.358	0.072	-0.32	-123.07	-62.402	13.649
297	-6.033	-6.033	0	-0.954	-19.043	-40.349	0.072	-0.182	-110.138	-59.392	19.534
298	-5.995	-5.995	0	-0.913	-17.309	-43.858	0.072	-0.288	-116.303	-61.167	13.627
299	-5.977	-5.977	0	-0.913	-16.772	-44.852	0.072	-0.297	-116.855	-61.624	13.073
300	-5.969	-5.969	0	-0.903	-15.863	-46.933	0.072	-0.345	-123.286	-62.796	15.312
301	-5.963	-5.963	0	-1.043	-13.14	-51.116	0.072	-0.16	-120.325	-64.256	11.091
302	-5.958	-5.958	-0.032	-0.919	-17.672	-51.257	0.072	-0.157	-129.051	-68.928	12.493
303	-5.921	-5.921	0	-0.892	-15.515	-51.379	0.072	-0.325	-124.672	-66.894	12.913
304	-5.899	-5.899	0	-1.011	-15.285	-45.258	0.072	-0.171	-116.291	-60.542	9.329
305	-5.87	-5.87	0	-1.077	-11.918	-55.374	0.072	-0.269	-124.653	-67.292	12.638

Compound **2**

	docking score	glide gscore	glide lipo	glide hbond	glide evdw	glide ecoul	glide erotb	glide esite	glide emodel	glide energy	glide einterna
1	-8.076	-8.076	-0.023	-1.518	-27.2	-64.047	0.095	-0.21	-233.573	-91.247	6.637
2	-7.379	-7.379	0	-1.635	-21.54	-69.86	0.095	-0.185	-217.627	-91.4	13.682
3	-7.372	-7.372	0	-1.679	-20.798	-69.435	0.095	-0.165	-215.512	-90.233	15.337
4	-7.369	-7.369	0	-1.693	-18.722	-76.895	0.095	-0.255	-221.296	-95.617	14.441
5	-7.228	-7.228	0	-1.699	-22.098	-61.565	0.095	-0.185	-204.4	-83.663	19.241
6	-7.214	-7.214	-0.038	-1.521	-23.537	-65.512	0.095	-0.193	-210.922	-89.049	16.465
7	-7.117	-7.117	-0.064	-1.248	-22.727	-64.818	0.095	-0.048	-186.263	-87.545	59.242
8	-7.077	-7.077	0	-1.555	-20.598	-67.576	0.095	-0.132	-207.941	-88.174	15.736
9	-7.033	-7.033	0	-1.26	-23.268	-58.644	0.095	-0.255	-200.507	-81.911	16.482
10	-6.976	-6.976	0	-1.181	-23.291	-63.167	0.095	-0.226	-207.2	-86.458	10.114
11	-6.933	-6.933	0	-1.649	-19.291	-65.669	0.095	-0.193	-201.265	-84.96	17.445
12	-6.92	-6.92	0	-1.378	-21.254	-62.306	0.095	-0.148	-201.627	-83.561	13.464
13	-6.855	-6.855	0	-1.452	-21.055	-67.179	0.095	-0.135	-202.47	-88.234	18.77
14	-6.315	-6.315	0	-0.977	-15.103	-57.727	0.095	-0.184	-177.886	-72.829	17.637
15	-6.173	-6.173	0	-0.761	-16.984	-50.317	0.095	-0.152	-174.38	-67.3	8.487
16	-6.145	-6.145	0	-1.044	-15.086	-54.677	0.095	-0.175	-174.34	-69.763	12.486
17	-6.035	-6.035	0	-1.138	-15.168	-53.674	0.095	-0.234	-170.918	-68.842	13.501
18	-5.966	-5.966	0	-1.098	-16.096	-55.775	0.095	-0.159	-171.351	-71.871	16.227

Compound **3**

	docking score	glide gscore	glide lipo	glide hbond	glide evdw	glide ecoul	glide erotb	glide esite	glide emodel	glide energy	glide einterna
1	-7.639	-7.639	0	-1.596	-22.353	-76.429	0.106	-0.357	-244.53	-98.781	11.887
2	-7.615	-7.615	0	-1.851	-21.728	-65.295	0.106	-0.15	-229.756	-87.023	19.312
3	-7.561	-7.561	0	-1.747	-18.388	-81.065	0.106	-0.195	-239.258	-99.453	22.989
4	-7.234	-7.234	0	-1.267	-21.054	-74.276	0.106	-0.346	-219.509	-95.33	21.591
5	-7.216	-7.216	-0.006	-1.702	-19.231	-84.807	0.106	-0.253	-251.892	-104.038	14.291
6	-7.18	-7.18	-0.022	-1.543	-18.126	-77.715	0.106	-0.215	-217.894	-95.841	23.833
7	-7.121	-7.121	0	-1.536	-13.987	-78.144	0.106	-0.279	-237.09	-92.131	23.461
8	-7.109	-7.109	0	-1.279	-24.737	-70.186	0.106	-0.249	-217.628	-94.923	19.626
9	-7.005	-7.005	0	-1.475	-18.675	-77.146	0.106	-0.253	-226.046	-95.821	30.299
10	-6.985	-6.985	0	-1.19	-18.207	-73.084	0.106	-0.316	-215.618	-91.291	11.992
11	-6.974	-6.974	-0.008	-1.554	-17.293	-80.587	0.106	-0.274	-238.086	-97.879	19.223
12	-6.869	-6.869	0	-1.388	-16.471	-81.471	0.106	-0.207	-227.783	-97.942	25.446
13	-6.751	-6.751	0	-1.173	-19.4	-79.968	0.106	-0.264	-237.175	-99.367	21.443
14	-6.717	-6.717	0	-1.337	-19.168	-72.093	0.106	-0.304	-211.663	-91.261	9.853
15	-6.698	-6.698	0	-1.34	-16.234	-91.033	0.106	-0.243	-239.715	-107.267	13.114
16	-6.634	-6.634	0	-1.502	-9.953	-95.159	0.106	-0.191	-239.981	-105.111	18.298
17	-6.633	-6.633	0	-1.248	-15.623	-73.914	0.106	-0.26	-210.907	-89.537	32.51
18	-6.602	-6.602	0	-1.404	-17.189	-93.191	0.106	-0.236	-246.729	-110.38	13.919
19	-6.596	-6.596	0	-1.346	-13.015	-90.64	0.106	-0.184	-229.591	-103.655	10.4
20	-6.583	-6.583	-0.024	-1.072	-24.703	-70.028	0.106	-0.133	-221.545	-94.731	19.526
21	-6.571	-6.571	0	-1.337	-14.922	-73.679	0.106	-0.214	-207.564	-88.601	23.352
22	-6.496	-6.496	0	-1.252	-16.037	-80.013	0.106	-0.299	-224.832	-96.05	24.446
23	-6.49	-6.49	0	-1.215	-16.9	-91.103	0.106	-0.192	-226.439	-108.003	21.192
24	-6.438	-6.438	0	-1.021	-16.075	-65.139	0.106	-0.269	-190.807	-81.214	20.828
25	-6.422	-6.422	0	-1.03	-15.805	-64.483	0.106	-0.258	-203.1	-80.287	9.411
26	-6.221	-6.221	0	-1.235	-9.35	-102.846	0.106	-0.146	-215.211	-112.196	52.097
27	-6.386	-6.386	0	-1.122	-13.119	-71.728	0.106	-0.264	-183.39	-84.847	56.532
28	-6.381	-6.381	0	-1.336	-8.565	-78.086	0.106	-0.273	-206.476	-86.651	14.071
29	-6.354	-6.354	0	-1.35	-8.788	-85.719	0.106	-0.153	-216.035	-94.508	20.591
30	-6.326	-6.326	0	-1.417	-11.981	-89.56	0.106	-0.191	-224.123	-101.54	29.486
31	-6.278	-6.278	0	-1.04	-15.496	-64.559	0.106	-0.345	-187.15	-80.056	21.879
32	-6.272	-6.272	0	-1.379	-12.703	-95.548	0.106	-0.218	-236.103	-108.251	20.277
33	-6.271	-6.271	0	-1.218	-10.938	-100.051	0.106	-0.15	-209.218	-110.99	54.546
34	-6.242	-6.242	0	-1.502	-7.146	-78.776	0.106	-0.265	-198.951	-85.922	8.663
35	-6.242	-6.242	0	-1.48	-7.373	-96.588	0.106	-0.15	-224.014	-103.961	18.988
36	-6.198	-6.198	0	-1.03	-12.161	-72.807	0.106	-0.216	-201.527	-84.968	24.074
37	-6.191	-6.191	0	-1.395	-12.315	-67.808	0.106	-0.286	-190.435	-80.123	10.389
38	-6.181	-6.181	0	-1.018	-15.914	-87.314	0.106	-0.139	-194.842	-103.229	62.269
39	-6.141	-6.141	0	-1.363	-9.704	-93.765	0.106	-0.175	-226.832	-103.469	26.07
40	-6.136	-6.136	0	-1.111	-14.363	-94.46	0.106	-0.188	-204.491	-108.823	36.278
41	-6.133	-6.133	0	-1.275	-16.068	-84.12	0.106	-0.048	-207.501	-100.187	14.027
42	-6.117	-6.117	0	-1.254	-11.168	-91.544	0.106	-0.185	-229.298	-102.713	15.998
43	-6.099	-6.099	0	-1.24	-16.824	-81.384	0.106	-0.124	-212.269	-98.208	24.986

44	-6.085	-6.085	0	-1.457	-6.209	-83.528	0.106	-0.199	-208.463	-89.737	23.958
45	-6.072	-6.072	0	-1.174	-16.088	-67.358	0.106	-0.2	-173.615	-83.446	47.97
46	-6.065	-6.065	0	-1.087	-18.494	-75.374	0.106	-0.134	-199.58	-93.868	27.497
47	-6.039	-6.039	0	-1.041	-7.832	-86.597	0.106	-0.264	-217.038	-94.429	5.461
48	-6.034	-6.034	0	-1.341	-8.412	-102.018	0.106	-0.123	-218.718	-110.43	33.777
49	-5.974	-5.974	0	-1.013	-18.686	-78.006	0.106	-0.133	-207.915	-96.692	16.667
50	-5.928	-5.928	0	-0.933	-8.116	-77.274	0.106	-0.246	-197.194	-85.39	9.923
51	-5.926	-5.926	0	-1.065	-5.265	-73.252	0.106	-0.254	-190.513	-78.517	11.637
52	-5.906	-5.906	0	-1.073	-4.61	-78.992	0.106	-0.259	-185.516	-83.602	15.26
53	-5.861	-5.861	0	-1.082	-12.8	-65.599	0.106	-0.246	-193.137	-78.399	13.741
54	-5.696	-5.696	0	-1.042	-5.378	-84.158	0.106	-0.266	-186.725	-89.536	17.978
55	-5.58	-5.58	0	-0.989	-9.18	-84.887	0.106	-0.238	-194.678	-94.067	30.283
56	-5.537	-5.537	0	-0.98	-9.855	-72.74	0.106	-0.17	-169.674	-82.595	34.044
57	-5.534	-5.534	0	-1.045	-8.781	-83.684	0.106	-0.156	-187.928	-92.464	27.067
58	-5.49	-5.49	0	-1.196	-3.526	-79.862	0.106	-0.224	-188.763	-83.388	30.841
59	-5.488	-5.488	0	-0.892	-9.116	-81.883	0.106	-0.246	-188.56	-90.999	32.582
60	-5.427	-5.427	0	-0.808	-9.366	-70.165	0.106	-0.257	-173.847	-79.531	12.666
61	-5.381	-5.381	-0.002	-0.703	-11.052	-64.849	0.106	-0.229	-164.584	-75.902	24.959
62	-5.043	-5.043	0	-0.25	-13.587	-69.014	0.106	-0.219	-173.044	-82.601	17.758

Compound **4**

	docking score	glide gscore	glide lipo	glide hbond	glide evdw	glide ecoul	glide erotb	glide esite	glide emodel	glide energy	glide einterna
1	-8.115	-8.115	0	-2.178	-19.239	-82.43	0.133	-0.285	-247.257	-101.669	17.165
2	-8.026	-8.026	0	-2.058	-20.152	-80.365	0.133	-0.248	-247.048	-100.516	11.901
3	-7.851	-7.851	0	-2.06	-15.174	-91.14	0.133	-0.204	-239.605	-106.314	31.727
4	-7.848	-7.848	0	-2.057	-15.398	-90.53	0.133	-0.203	-238.801	-105.928	32.452
5	-7.806	-7.806	-0.081	-1.576	-18.46	-81.913	0.133	-0.277	-234.974	-100.373	27.389
6	-7.501	-7.501	-0.051	-1.566	-18.011	-82.977	0.133	-0.249	-231.164	-100.988	24.631
7	-7.437	-7.437	0	-1.761	-15.224	-81.458	0.133	-0.268	-220.906	-96.681	34.101
8	-7.374	-7.374	0	-1.736	-14.715	-81.155	0.133	-0.262	-219.389	-95.87	33.154
9	-7.328	-7.328	0	-1.619	-21.492	-64.191	0.133	-0.141	-217.817	-85.683	14.162
10	-7.304	-7.304	0	-1.655	-16.444	-87.805	0.133	-0.228	-230.479	-104.249	25.066
11	-7.248	-7.248	0	-1.735	-16.598	-69.701	0.133	-0.286	-217.233	-86.299	13.532
12	-7.187	-7.187	0	-1.744	-13.149	-83.001	0.133	-0.237	-214.176	-96.151	37.005
13	-7.113	-7.113	0	-1.791	-12.867	-72.132	0.133	-0.296	-208.421	-84.998	23.423
14	-7.091	-7.091	0	-1.335	-22.646	-55.859	0.133	-0.215	-205.299	-78.504	15.833
15	-6.775	-6.775	0	-1.493	-20.762	-67.827	0.133	-0.143	-211.471	-88.589	11.638
16	-6.698	-6.698	-0.029	-1.068	-16.875	-57.262	0.133	-0.19	-192.557	-74.137	17.645
17	-6.677	-6.677	-0.035	-1.331	-18.749	-60.64	0.133	-0.149	-196.454	-79.389	19.574
18	-6.527	-6.527	0	-1.16	-21.302	-62.84	0.133	-0.21	-201.214	-84.142	13.835
19	-6.368	-6.368	0	-1.03	-20.465	-56.41	0.133	-0.223	-191.63	-76.874	12.441

Compound **5**

	docking score	glide gscore	glide lipo	glide hbond	glide evdw	glide ecoul	glide erotb	glide esite	glide emodel	glide energy	glide einterna
1	-9.23	-9.23	-0.009	-1.636	-21.012	-41.315	0.071	-0.413	-157.784	-62.327	9.159
2	-9.168	-9.168	-0.004	-1.73	-18.674	-42.088	0.071	-0.386	-155.077	-60.762	10.099
3	-8.808	-8.808	-0.055	-1.241	-22.355	-31.241	0.071	-0.325	-138.668	-53.597	17.035
4	-8.807	-8.807	-0.019	-1.489	-17.603	-40.501	0.071	-0.368	-142.199	-58.104	18.966
5	-8.805	-8.805	-0.007	-1.771	-19.938	-43.508	0.071	-0.438	-153.468	-63.445	10.815
6	-8.707	-8.707	-0.017	-1.402	-17.098	-41.829	0.071	-0.383	-140.728	-58.926	21.437
7	-8.699	-8.699	0	-1.524	-14.359	-44.422	0.071	-0.409	-147.211	-58.781	11.71
8	-8.688	-8.688	-0.005	-1.704	-19.284	-43.256	0.071	-0.423	-151.272	-62.54	10.854
9	-8.681	-8.681	0	-1.429	-16.516	-42.941	0.071	-0.375	-141.653	-59.456	20.082
10	-8.663	-8.663	-0.004	-1.758	-18.119	-43.367	0.071	-0.408	-150.134	-61.486	10.494
11	-8.551	-8.551	-0.009	-1.631	-18.17	-41.6	0.071	-0.412	-147.648	-59.77	9.599
12	-8.352	-8.352	-0.002	-1.567	-16.679	-45.413	0.071	-0.383	-146.972	-62.092	11.289
13	-8.297	-8.297	-0.049	-1.168	-21.874	-34.409	0.071	-0.412	-142.764	-56.282	6.885
14	-8.136	-8.136	-0.037	-1.11	-20.516	-31.029	0.071	-0.4	-134.918	-51.545	9.609
15	-8.091	-8.091	0	-1.406	-14.103	-41.843	0.071	-0.433	-136.967	-55.946	13.335
16	-8.048	-8.048	-0.133	-1.258	-19.866	-33.711	0.071	-0.368	-135.671	-53.578	10.258
17	-7.98	-7.98	-0.223	-0.85	-25.338	-30.185	0.071	-0.272	-130.257	-55.523	20.128
18	-7.954	-7.954	0	-1.477	-10.778	-42.185	0.071	-0.392	-135.424	-52.963	7.48
19	-7.828	-7.828	0	-1.388	-14.451	-52.22	0.071	-0.317	-144.876	-66.67	17.486
20	-7.802	-7.802	-0.01	-1.048	-20.907	-33.772	0.071	-0.42	-135.678	-54.679	7.113
21	-7.8	-7.8	-0.232	-0.974	-25.029	-29.992	0.071	-0.257	-128.101	-55.021	19.598
22	-7.786	-7.786	-0.02	-1.085	-18.91	-42.073	0.071	-0.313	-135.459	-60.983	23.996
23	-7.7	-7.7	-0.237	-0.938	-23.67	-31.824	0.071	-0.255	-126.667	-55.494	21.287
24	-7.662	-7.662	-0.027	-1.093	-23.549	-30.541	0.071	-0.383	-132.442	-54.09	9.386
25	-7.633	-7.633	-0.128	-1.066	-16.436	-31.905	0.071	-0.333	-126.155	-48.342	9.819
26	-7.624	-7.624	-0.074	-0.929	-20.814	-35.443	0.071	-0.31	-128.535	-56.258	17.468
27	-7.586	-7.586	-0.031	-1.115	-16.641	-37.702	0.071	-0.353	-125.714	-54.342	18.463
28	-7.457	-7.457	-0.221	-0.955	-24.065	-29.317	0.071	-0.241	-124.643	-53.381	15.947
29	-7.353	-7.353	-0.009	-1.179	-21.949	-44.53	0.102	-0.263	-140.043	-66.479	12.25
30	-7.333	-7.333	-0.05	-1.019	-13.095	-34.45	0.071	-0.336	-122.259	-47.545	9.532
31	-7.315	-7.315	0	-1.138	-22.84	-42.466	0.102	-0.275	-139.018	-65.306	11.125
32	-7.303	-7.303	0	-1.364	-14.864	-48.834	0.071	-0.237	-142.434	-63.698	4.746
33	-7.285	-7.285	-0.008	-0.975	-19.953	-40.146	0.071	-0.151	-134.934	-60.099	12.146
34	-7.251	-7.251	0	-1.401	-13.413	-47.861	0.071	-0.22	-138.808	-61.274	6.006
35	-7.127	-7.127	0	-1.043	-26.244	-43.574	0.071	-0.305	-139.9	-69.818	18.162
36	-7.095	-7.095	-0.359	-1.094	-22.839	-39.995	0.102	-0.302	-130.812	-62.834	14.819
37	-7.074	-7.074	0	-0.76	-13.594	-41.229	0.071	-0.381	-126.65	-54.823	13.483
38	-7.01	-7.01	-0.326	-1.097	-22.027	-37.894	0.102	-0.326	-127.789	-59.921	13.232
39	-7.008	-7.008	0	-1.247	-19.015	-37.201	0.071	-0.31	-125.107	-56.216	17.888
40	-6.976	-6.976	-0.239	-1.259	-20.186	-44.218	0.102	-0.326	-130.783	-64.405	15.483
41	-6.965	-6.965	-0.228	-1.112	-22.765	-34.157	0.102	-0.254	-121.668	-56.922	18.521
42	-6.955	-6.955	0	-1.362	-16.72	-41.059	0.071	-0.288	-128.716	-57.78	12.623
43	-6.941	-6.941	0	-0.999	-24.015	-44.791	0.071	-0.271	-141.239	-68.806	9.312

44	-6.934	-6.934	-0.013	-1.022	-21.345	-42.519	0.102	-0.299	-132.82	-63.864	16.119
45	-6.923	-6.923	-0.211	-1.206	-22.151	-44.624	0.102	-0.432	-129.958	-66.776	20.75
46	-6.897	-6.897	-0.003	-1.217	-16.58	-39.807	0.071	-0.331	-127.356	-56.387	11.263
47	-6.885	-6.885	0	-0.942	-19.216	-42.209	0.102	-0.294	-126.926	-61.424	15.312
48	-6.882	-6.882	0	-1.24	-17.773	-35.252	0.071	-0.278	-121.495	-53.025	15.938
49	-6.879	-6.879	0	-1.074	-19.74	-36.304	0.071	-0.297	-124.393	-56.045	16.115
50	-6.878	-6.878	0	-0.996	-22.858	-47.174	0.071	-0.275	-140.454	-70.031	11.944
51	-6.87	-6.87	0	-1.127	-18.98	-36.745	0.071	-0.294	-123.444	-55.726	17.179
52	-6.866	-6.866	-0.046	-1.234	-17.321	-39.697	0.071	-0.25	-124.037	-57.018	18.487
53	-6.814	-6.814	0	-1.185	-16.726	-37.462	0.071	-0.291	-123.898	-54.188	11.95
54	-6.786	-6.786	0	-1.04	-19.864	-40.432	0.071	-0.287	-132.128	-60.296	7.075
55	-6.777	-6.777	0	-0.966	-21.121	-43.498	0.071	-0.284	-134.144	-64.619	11.482
56	-6.761	-6.761	0	-1.098	-18.026	-46.346	0.071	-0.289	-130.376	-64.372	18.169
57	-6.743	-6.743	-0.172	-1.501	-16.333	-48.002	0.102	-0.355	-129.681	-64.335	12.608
58	-6.692	-6.692	0	-0.558	-11.809	-40.596	0.071	-0.29	-119.488	-52.406	14.492
59	-6.658	-6.658	-0.004	-1.158	-13.794	-40.739	0.071	-0.348	-124.812	-54.533	7.347
60	-6.658	-6.658	-0.004	-1.181	-13.167	-41.765	0.071	-0.355	-125.763	-54.932	6.241
61	-6.651	-6.651	0	-0.453	-13.285	-41.178	0.071	-0.28	-121.234	-54.463	14.198
62	-6.65	-6.65	-0.153	-1.01	-24.127	-35.588	0.102	-0.355	-124.64	-59.715	11.468
63	-6.649	-6.649	-0.004	-1.183	-12.98	-41.235	0.071	-0.353	-125.28	-54.216	5.568
64	-6.646	-6.646	-0.076	-1.464	-16.775	-45.724	0.102	-0.369	-127.453	-62.499	11.323
65	-6.644	-6.644	-0.031	-0.936	-12.396	-33.935	0.071	-0.3	-113.338	-46.33	9.999
66	-6.639	-6.639	0	-1.086	-16.053	-36.26	0.071	-0.284	-120.532	-52.313	11.034
67	-6.624	-6.624	-0.135	-1.223	-15.175	-53.141	0.102	-0.384	-133.007	-68.315	11.383
68	-6.549	-6.549	-0.058	-1.216	-14.96	-47.426	0.102	-0.402	-126.868	-62.385	10.201
69	-6.544	-6.544	-0.097	-1.206	-21.056	-41.143	0.102	-0.289	-127.322	-62.199	8.827
70	-6.529	-6.529	0	-1.056	-14.062	-35.445	0.071	-0.311	-114.088	-49.507	15.872
71	-6.484	-6.484	0	-0.832	-17.529	-42.712	0.102	-0.378	-116.92	-60.241	24.438
72	-6.48	-6.48	0	-0.865	-17.526	-43.465	0.071	-0.267	-128.861	-60.991	8.195
73	-6.462	-6.462	0	-1.205	-15.178	-41.258	0.102	-0.531	-116.806	-56.436	16.585
74	-6.448	-6.448	0	-1.012	-14.283	-41.867	0.071	-0.263	-122.476	-56.15	10.581
75	-6.434	-6.434	-0.045	-1.166	-23.699	-28.767	0.102	-0.119	-118.332	-52.466	7.737
76	-6.396	-6.396	0	-1.081	-22.823	-41.686	0.102	-0.247	-124.307	-64.509	16.323
77	-6.387	-6.387	-0.016	-1.167	-20.214	-41.108	0.102	-0.263	-122.705	-61.322	19.121
78	-6.376	-6.376	-0.062	-1.169	-13.421	-47.527	0.102	-0.35	-123.901	-60.948	9.583
79	-6.372	-6.372	-0.076	-0.887	-25.756	-27.409	0.102	-0.132	-115.865	-53.165	12.719
80	-6.364	-6.364	0	-0.723	-23.47	-36.532	0.102	-0.287	-118.99	-60.002	23.394
81	-6.362	-6.362	-0.002	-1.195	-16.196	-40.827	0.102	-0.232	-118.399	-57.023	12.46
82	-6.362	-6.362	-0.177	-1.094	-17.712	-42.799	0.102	-0.29	-122.022	-60.51	12.187
83	-6.324	-6.324	-0.107	-0.915	-25.67	-28.26	0.102	-0.116	-116.469	-53.93	12.005
84	-6.304	-6.304	0	-0.824	-14.608	-33.102	0.071	-0.291	-111.38	-47.71	12.683
85	-6.287	-6.287	-0.048	-1.216	-24.512	-24.925	0.102	-0.131	-113.841	-49.437	7.469
86	-6.25	-6.25	-0.04	-1.026	-14.828	-38.204	0.102	-0.32	-114.19	-53.032	10.512
87	-6.228	-6.228	-0.029	-0.943	-16.906	-40.495	0.102	-0.287	-117.687	-57.401	17.78
88	-6.174	-6.174	-0.172	-0.877	-18.669	-36.916	0.102	-0.292	-115.642	-55.585	11.09
89	-6.163	-6.163	0	-0.692	-14.992	-38.516	0.102	-0.373	-115.955	-53.508	12.015
90	-6.128	-6.128	0	-0.581	-15.932	-41.484	0.071	-0.292	-118.144	-57.417	14.679
91	-6.118	-6.118	-0.118	-1.183	-27.658	-22.472	0.102	-0.107	-109.588	-50.131	13.703
92	-6.114	-6.114	0	-1.019	-17.51	-42.576	0.102	-0.321	-117.955	-60.087	16.787
93	-6.09	-6.09	-0.006	-0.833	-18.084	-32.433	0.102	-0.401	-110.816	-50.517	8.819

94	-6.063	-6.063	-0.004	-0.937	-20.049	-27.46	0.102	-0.207	-108.283	-47.509	9.877
95	-6.062	-6.062	-0.005	-0.931	-16.871	-33.456	0.102	-0.38	-110.437	-50.327	8.61
96	-6.04	-6.04	-0.002	-0.561	-17.143	-34.775	0.102	-0.261	-113.321	-51.918	8.105
97	-6.025	-6.025	0	-0.97	-13.916	-42.913	0.102	-0.461	-116.134	-56.828	9.438
98	-6.017	-6.017	-0.003	-0.61	-20.297	-43.153	0.102	-0.263	-123.937	-63.45	9.442
99	-6.011	-6.011	0	-0.611	-13.102	-40.012	0.071	-0.286	-113.736	-53.114	12.288
100	-5.998	-5.998	-0.07	-1.046	-13.653	-42.68	0.102	-0.302	-115.537	-56.333	9.059
101	-5.979	-5.979	-0.002	-0.885	-18.882	-40.095	0.102	-0.208	-114.691	-58.978	15.632
102	-5.867	-5.867	-0.018	-0.827	-17.597	-40.739	0.102	-0.204	-114.655	-58.336	12.036
103	-5.859	-5.859	0	-0.732	-20.574	-32.967	0.102	-0.2	-112.418	-53.541	6.76
104	-5.82	-5.82	-0.245	-0.883	-21.014	-23.772	0.102	-0.073	-104.29	-44.786	7.144
105	-5.807	-5.807	-0.242	-0.877	-20.661	-23.755	0.102	-0.076	-102.269	-44.416	10.172
106	-5.752	-5.752	-0.095	-0.781	-23.151	-24.267	0.102	-0.111	-103.685	-47.419	12.137
107	-5.689	-5.689	-0.051	-0.713	-25.627	-23.922	0.102	-0.102	-105.462	-49.55	11.499
108	-5.593	-5.593	-0.093	-0.829	-11.093	-28.939	0.102	-0.199	-92.517	-40.032	16.261
109	-5.575	-5.575	-0.094	-0.735	-22.693	-23.784	0.102	-0.106	-101.969	-46.477	9.859
110	-5.518	-5.518	-0.047	-0.908	-23.869	-22.198	0.102	-0.117	-102.593	-46.067	6.56
111	-5.516	-5.516	-0.032	-0.828	-21.383	-23.834	0.102	-0.113	-102.51	-45.217	4.97
112	-5.457	-5.457	-0.025	-0.985	-21.496	-22.393	0.102	-0.115	-100.471	-43.889	5.119
113	-5.448	-5.448	-0.022	-0.662	-13.1	-35.841	0.102	-0.211	-98.425	-48.94	19.125

Compound **6**

	docking score	glide gscore	glide lipo	glide hbond	glide evdw	glide ecoul	glide erotb	glide esite	glide emodel	glide energy	glide einterna
					40						
1	-8.885	-8.885	0	-1.353	-21.369	-39.75	0.093	-0.36	-149.661	-61.119	15.485
2	-8.62	-8.62	-0.009	-1.253	-24.704	-44.583	0.093	-0.279	-150.121	-69.287	16.346
3	-8.575	-8.575	-0.034	-1.173	-19.3	-31.024	0.093	-0.375	-117.409	-50.324	32.278
4	-8.448	-8.448	-0.045	-1.393	-22.68	-41.449	0.093	-0.379	-148.952	-64.129	13.469
5	-8.388	-8.388	0	-1.296	-21.343	-44.333	0.093	-0.293	-144.529	-65.676	15.525
6	-8.385	-8.385	-0.208	-0.878	-28.551	-28.294	0.093	-0.278	-131.559	-56.845	13.33
7	-8.371	-8.371	-0.045	-1.29	-23.349	-42.521	0.093	-0.372	-150.571	-65.87	12.037
8	-8.261	-8.261	-0.064	-1.288	-20.853	-38.099	0.093	-0.362	-133.358	-58.952	21.785
9	-8.187	-8.187	-0.002	-1.313	-19.984	-41.311	0.093	-0.376	-142.725	-61.295	14.599
10	-8.09	-8.09	0	-1.34	-17.776	-43.709	0.093	-0.364	-142.441	-61.485	13.432
11	-8.084	-8.084	-0.06	-1.07	-22.391	-39.267	0.093	-0.336	-142.775	-61.658	12.995
12	-8.024	-8.024	-0.001	-1.164	-20.287	-39.059	0.093	-0.348	-140.401	-59.346	11.805
13	-7.97	-7.97	-0.072	-1.04	-20.59	-33.951	0.093	-0.332	-126.605	-54.541	20.474
14	-7.845	-7.845	-0.158	-0.839	-24.2	-32.64	0.093	-0.354	-118.316	-56.84	29.315
15	-7.801	-7.801	0	-0.917	-11.73	-35.024	0.093	-0.272	-125.635	-46.754	11.329
16	-7.544	-7.544	-0.108	-0.874	-20.754	-27.282	0.093	-0.243	-117.751	-48.036	7.008
17	-7.519	-7.519	-0.201	-0.96	-17.752	-29.31	0.093	-0.239	-112.456	-47.063	15.159
18	-7.038	-7.038	-0.01	-1.689	-25.089	-40.39	0.093	-0.415	-132.689	-65.479	18.115
19	-7.029	-7.029	0	-1.026	-21.289	-41.647	0.133	-0.346	-129.005	-62.937	13.015
20	-7.019	-7.019	-0.058	-1.361	-25.089	-41.945	0.133	-0.386	-117.104	-67.034	36.107
21	-6.999	-6.999	0	-0.715	-13.593	-33.484	0.093	-0.373	-113.667	-47.077	18.522
22	-6.962	-6.962	-0.045	-1.362	-19.661	-37.121	0.133	-0.45	-119.949	-56.782	17.442
23	-6.952	-6.952	-0.084	-0.808	-13.827	-26.026	0.093	-0.233	-107.554	-39.853	8.163
24	-6.95	-6.95	0	-1.023	-23.162	-40.267	0.093	-0.122	-132.199	-63.429	13.099
25	-6.945	-6.945	-0.049	-1.403	-18.614	-43.07	0.133	-0.435	-124.039	-61.684	18.711
26	-6.941	-6.941	-0.045	-1.539	-14.093	-40.749	0.133	-0.498	-121.35	-54.843	10.322
27	-6.87	-6.87	-0.051	-1.469	-20.081	-43.779	0.133	-0.443	-125.159	-63.86	19.289
28	-6.772	-6.772	-0.042	-1.221	-19.056	-39.722	0.133	-0.3	-115.044	-58.777	18.905
29	-6.769	-6.769	0	-1.095	-17.246	-39.265	0.093	-0.348	-120.823	-56.511	18.082
30	-6.753	-6.753	-0.019	-1.05	-18.115	-34.427	0.093	-0.341	-117.627	-52.542	16.199
31	-6.735	-6.735	0	-0.992	-19.171	-35.408	0.093	-0.347	-119.756	-54.579	15.615
32	-6.705	-6.705	-0.002	-1.283	-17.034	-43.028	0.133	-0.242	-124.039	-60.062	10.509
33	-6.689	-6.689	-0.021	-0.709	-12.498	-25.78	0.093	-0.235	-95.079	-38.278	16.229
34	-6.68	-6.68	-0.049	-1.364	-19.803	-43.845	0.133	-0.381	-123.768	-63.648	17.714
35	-6.631	-6.631	-0.053	-1.323	-16.221	-37.234	0.133	-0.512	-116.198	-53.455	11.462
36	-6.626	-6.626	-0.36	-1.039	-24.44	-32.491	0.133	-0.112	-108.715	-56.931	25.051
37	-6.625	-6.625	-0.165	-1.089	-19.281	-36.486	0.133	-0.299	-112.434	-55.767	23.491
38	-6.617	-6.617	-0.065	-1.008	-24.902	-40.034	0.133	-0.201	-123.497	-64.936	19.54
39	-6.598	-6.598	0	-1.024	-15.894	-34.95	0.093	-0.335	-114.32	-50.844	16.064
40	-6.597	-6.597	-0.008	-1.357	-21.783	-31.381	0.133	-0.139	-100.012	-53.164	34.353
41	-6.594	-6.594	-0.054	-1.293	-19.708	-39.562	0.133	-0.333	-117.638	-59.269	11.254
42	-6.547	-6.547	-0.046	-1.355	-18.472	-38.425	0.133	-0.327	-115.465	-56.897	18.064
43	-6.532	-6.532	0	-0.972	-22.401	-37.683	0.133	-0.349	-121.165	-60.084	12.745

44	-6.49	-6.49	-0.047	-1.217	-20.3	-37.424	0.133	-0.324	-114.281	-57.724	20.925
45	-6.425	-6.425	-0.021	-1.374	-19.985	-35.548	0.133	-0.13	-116.908	-55.533	9.939
46	-6.421	-6.421	-0.053	-1.053	-21.843	-33.581	0.133	-0.33	-113.778	-55.424	15.901
47	-6.406	-6.406	-0.199	-0.893	-26.66	-28.984	0.133	-0.101	-109.151	-55.644	17.33
48	-6.395	-6.395	-0.046	-0.886	-15.383	-30.873	0.133	-0.103	-110.353	-46.256	10.422
49	-6.387	-6.387	0	-0.86	-21.766	-41.485	0.133	-0.347	-123.677	-63.251	11.055
50	-6.335	-6.335	-0.011	-1.307	-21.284	-41.799	0.093	-0.409	-123.257	-63.083	16.962
51	-6.334	-6.334	-0.168	-0.941	-21.128	-28.395	0.133	-0.302	-98.237	-49.522	25.512
52	-6.321	-6.321	-0.092	-0.93	-23.462	-30.808	0.133	-0.156	-106.788	-54.27	17.659
53	-6.309	-6.309	-0.134	-1.011	-23.895	-31.504	0.133	-0.103	-105.43	-55.398	22.399
54	-6.303	-6.303	-0.043	-1.21	-18.973	-33.997	0.133	-0.235	-110.956	-52.971	20.649
55	-6.297	-6.297	-0.185	-0.95	-18.702	-27.209	0.133	-0.356	-93.228	-45.911	27.582
56	-6.282	-6.282	-0.05	-1.003	-17.451	-35.255	0.133	-0.443	-112.554	-52.706	10.037
57	-6.281	-6.281	-0.007	-0.86	-16.83	-42.764	0.133	-0.206	-112.895	-59.594	15.304
58	-6.268	-6.268	-0.062	-0.958	-25.005	-26.583	0.133	-0.123	-113.287	-51.587	12.454
59	-6.246	-6.246	0	-0.961	-19.001	-42.03	0.133	-0.188	-105.184	-61.031	32.94
60	-6.197	-6.197	0	-1.052	-17.613	-41.544	0.133	-0.154	-119.647	-59.158	13.349
61	-6.187	-6.187	-0.003	-0.619	-19.934	-26.634	0.133	-0.285	-105.031	-46.568	17.164
62	-6.185	-6.185	0	-0.764	-22.198	-35.122	0.133	-0.174	-117.079	-57.319	14.539
63	-6.184	-6.184	-0.064	-0.87	-19.25	-27.666	0.133	-0.196	-93.922	-46.916	26.025
64	-6.177	-6.177	0	-1.057	-13.969	-30.316	0.133	-0.33	-100.934	-44.285	14.271
65	-6.162	-6.162	-0.248	-0.699	-26.881	-25.707	0.133	-0.104	-101.739	-52.588	21.297
66	-6.15	-6.15	-0.14	-0.861	-14.696	-38.411	0.133	-0.322	-111.776	-53.107	15.957
67	-6.147	-6.147	-0.12	-0.886	-26.657	-25.503	0.133	-0.116	-107.244	-52.16	16.779
68	-6.11	-6.11	0	-1.251	-19.06	-40.882	0.093	-0.358	-117.703	-59.942	16.905
69	-6.105	-6.105	-0.111	-0.994	-20.552	-28.779	0.133	-0.105	-104.68	-49.331	15.37
70	-6.097	-6.097	0	-0.983	-13.547	-30.99	0.133	-0.345	-105.888	-44.538	9.451
71	-6.086	-6.086	-0.109	-0.917	-23.17	-26.142	0.133	-0.092	-99.946	-49.312	16.855
72	-6.079	-6.079	-0.059	-0.581	-19.693	-25.413	0.093	-0.205	-96.926	-45.106	14.353
73	-6.06	-6.06	-0.181	-0.911	-25.886	-24.486	0.133	-0.102	-99.688	-50.372	18.987
74	-6.057	-6.057	-0.144	-0.728	-21.934	-27.096	0.133	-0.174	-108.485	-49.03	12.365
75	-6.047	-6.047	-0.117	-0.588	-26.733	-27.335	0.133	-0.126	-109.592	-54.069	13.827
76	-6.045	-6.045	-0.107	-1.043	-18.12	-32.006	0.133	-0.104	-98.126	-50.126	21.325
77	-6.04	-6.04	-0.117	-0.735	-25.356	-26.161	0.133	-0.122	-110.14	-51.517	13.667
78	-6.038	-6.038	0	-0.798	-11.401	-29.534	0.093	-0.234	-88.927	-40.936	28.715
79	-6.038	-6.038	-0.063	-0.905	-20.684	-27.493	0.133	-0.169	-108.034	-48.177	11.149
80	-6.035	-6.035	0	-1.156	-17.64	-38.418	0.093	-0.382	-111.244	-56.058	20.432
81	-6.026	-6.026	-0.014	-1.19	-18.699	-40.623	0.093	-0.381	-115.866	-59.322	17.522
82	-5.977	-5.977	0	-1.079	-14.086	-34.342	0.133	-0.327	-102.663	-48.427	14.967
83	-5.955	-5.955	0	-0.582	-18.416	-35.371	0.133	-0.361	-109.125	-53.787	12.312
84	-5.954	-5.954	0	-1.058	-13.633	-34.036	0.133	-0.316	-102.655	-47.668	12.997
85	-5.935	-5.935	0	-0.93	-16.013	-33.237	0.133	-0.337	-99.382	-49.25	14.928
86	-5.915	-5.915	-0.049	-0.587	-24.163	-26.849	0.133	-0.177	-99.06	-51.012	18.715
87	-5.912	-5.912	0	-0.844	-17.914	-28.551	0.133	-0.306	-94.518	-46.465	18.653
88	-5.903	-5.903	-0.02	-0.907	-18.258	-42.784	0.133	-0.197	-110.932	-61.042	14.799
89	-5.894	-5.894	-0.005	-0.788	-9.031	-27.048	0.093	-0.213	-89.571	-36.079	14.734
90	-5.86	-5.86	0	-0.61	-11.597	-27.28	0.093	-0.233	-89.708	-38.876	12.071
91	-5.859	-5.859	-0.022	-0.519	-20.292	-35.241	0.133	-0.211	-105.93	-55.533	20.199
92	-5.793	-5.793	-0.06	-0.796	-25.145	-24.45	0.133	-0.138	-105.944	-49.595	12.873
93	-5.77	-5.77	0	-0.76	-18.21	-38.178	0.133	-0.233	-112.511	-56.388	12.821

94	-5.757	-5.757	-0.018	-0.58	-16.412	-27.199	0.133	-0.256	-97.586	-43.611	10.951
95	-5.753	-5.753	-0.209	-0.566	-19.329	-26.995	0.133	-0.133	-96.426	-46.324	11.457
96	-5.716	-5.716	-0.032	-0.921	-13.397	-31.288	0.133	-0.226	-87.881	-44.685	31.646
97	-5.715	-5.715	-0.045	-0.769	-10.566	-25.004	0.093	-0.185	-88.176	-35.57	12.806
98	-5.693	-5.693	-0.029	-0.692	-19.533	-27.978	0.133	-0.128	-98.923	-47.51	7.685
99	-5.682	-5.682	-0.105	-0.721	-15.862	-26.612	0.133	-0.195	-85.051	-42.474	25.142
100	-5.673	-5.673	-0.009	-0.911	-13.527	-29.689	0.133	-0.211	-89.92	-43.216	16.715
101	-5.668	-5.668	-0.085	-0.564	-24.129	-25.204	0.133	-0.129	-93.821	-49.333	21.049
102	-5.653	-5.653	-0.021	-0.767	-14.73	-27.687	0.133	-0.173	-91.76	-42.418	11.036
103	-5.642	-5.642	-0.021	-0.813	-15.961	-32.508	0.133	-0.143	-89.486	-48.468	27.48
104	-5.594	-5.594	-0.009	-0.735	-13.6	-26.02	0.133	-0.4	-85.47	-39.62	23.826
105	-5.583	-5.583	0	-0.469	-8.772	-37.778	0.093	-0.238	-94.599	-46.55	12.269
106	-5.574	-5.574	-0.079	-0.76	-14.063	-27.833	0.133	-0.165	-96.308	-41.896	11.985
107	-5.573	-5.573	0	-0.583	-12.555	-28.917	0.133	-0.27	-93.625	-41.472	16.483
108	-5.553	-5.553	-0.009	-0.445	-12.991	-25.431	0.093	-0.199	-91.048	-38.423	9.431
109	-5.53	-5.53	-0.007	-0.544	-12.488	-28.819	0.133	-0.264	-93.59	-41.308	15.297
110	-5.521	-5.521	-0.099	-0.643	-24.845	-23.562	0.133	-0.114	-103.013	-48.408	10.46
111	-5.509	-5.509	-0.099	-0.562	-27.626	-23.028	0.133	-0.109	-102.892	-50.653	14.928
112	-5.398	-5.398	-0.013	-0.468	-22.178	-24.964	0.133	-0.098	-98.66	-47.142	13.965
113	-5.353	-5.353	0	-0.433	-11.717	-24.696	0.093	-0.193	-80.932	-36.412	21.506
114	-5.342	-5.342	-0.167	-0.526	-26.789	-20.932	0.133	-0.138	-101.235	-47.721	8.751
115	-5.304	-5.304	-0.136	-0.659	-25.615	-21.411	0.133	-0.1	-90.672	-47.026	15.66
116	-5.279	-5.279	-0.13	-0.489	-18.14	-24.409	0.133	-0.178	-84.253	-42.549	25.607
117	-4.903	-4.903	0	-0.374	-13.834	-25.12	0.133	-0.202	-80.266	-38.954	18.633
118	-4.659	-4.659	-0.101	-0.624	-23.349	-18.476	0.133	-0.1	-79.101	-41.824	15.874
119	-4.446	-4.446	-0.02	-0.728	-15.809	-19.309	0.133	-0.145	-66.369	-35.118	23.794

Compound **7**

	docking score	glide gscore	glide lipo	glide hbond	glide evdw	glide ecoul	glide erotb	glide esite	glide emodel	glide energy	glide einterna
1	-7.661	-7.661	-0.024	-0.855	-17.812	-39.683	0.099	-0.4	-121.05	-57.495	25.039
2	-7.641	-7.641	0	-0.782	-9.172	-35.054	0.099	-0.38	-112.321	-44.226	15.569
3	-7.445	-7.445	-0.024	-0.964	-14.188	-34.904	0.099	-0.256	-115.474	-49.092	13.935
4	-7.393	-7.393	-0.025	-0.605	-11.29	-26.6	0.099	-0.187	-100.981	-37.889	20.641
5	-7.101	-7.101	-0.013	-0.956	-16.721	-39.706	0.099	-0.375	-117.828	-56.428	18.225
6	-7.067	-7.067	-0.172	-1.454	-22.254	-47.526	0.141	-0.434	-118.728	-69.779	31.934
7	-6.74	-6.74	-0.022	-1.232	-20.653	-47.254	0.141	-0.195	-127.723	-67.907	13.175
8	-6.711	-6.711	-0.001	-0.47	-18.732	-24.301	0.099	-0.183	-102.797	-43.033	12.717
9	-6.614	-6.614	-0.036	-0.616	-16.144	-26.933	0.099	-0.234	-98.068	-43.077	21.389
10	-6.609	-6.609	-0.032	-1.093	-20.507	-44.436	0.141	-0.198	-123.097	-64.942	13.914
11	-6.574	-6.574	0	-1.411	-6.466	-35.791	0.141	-0.256	-100.929	-42.258	12.193
12	-6.567	-6.567	0	-1.351	-3.427	-44.472	0.099	-0.388	-101.358	-47.899	23.502
13	-6.554	-6.554	-0.129	-0.4	-25.513	-21.278	0.099	-0.197	-105.782	-46.791	11.156
14	-6.444	-6.444	-0.008	-0.59	-14.054	-29.854	0.099	-0.223	-101.873	-43.909	12.065
15	-6.379	-6.379	0	-1.342	-4.611	-38.989	0.099	-0.376	-92.845	-43.6	28.2
16	-6.293	-6.293	0	-1.231	-5.08	-35.719	0.099	-0.377	-91.169	-40.799	24.239
17	-6.262	-6.262	0	-1.281	-10.103	-37.872	0.141	-0.203	-105.249	-47.975	8.84
18	-6.236	-6.236	0	-1.154	-5.971	-33.908	0.099	-0.353	-91.073	-39.879	21.474
19	-6.235	-6.235	-0.213	-0.904	-20.283	-27.101	0.141	-0.206	-104.69	-47.384	9.221
20	-6.21	-6.21	-0.004	-0.937	-16.244	-41.171	0.141	-0.36	-112.611	-57.416	11.983
21	-6.199	-6.199	-0.001	-0.673	-9.714	-24.198	0.099	-0.184	-87.279	-33.912	16.393
22	-6.188	-6.188	0	-1.343	-13.87	-42.047	0.141	-0.205	-109.611	-55.917	14.546
23	-6.159	-6.159	0	-1.353	-13.264	-41.96	0.141	-0.203	-107.975	-55.224	15.852
24	-6.15	-6.15	0	-0.999	-7.571	-28.274	0.099	-0.341	-87.36	-35.845	19.116
25	-6.15	-6.15	-0.123	-0.847	-20.602	-29.755	0.141	-0.217	-106.172	-50.356	10.507
26	-6.094	-6.094	0	-1.31	-5.847	-42.187	0.141	-0.21	-101.989	-48.034	12.167
27	-6.026	-6.026	-0.028	-0.985	-13.861	-32.995	0.141	-0.462	-101.377	-46.856	9.696
28	-6.009	-6.009	0	-0.567	-5.477	-24.977	0.099	-0.196	-80.902	-30.454	18.458
29	-5.997	-5.997	0	-1.237	-4.967	-39.852	0.141	-0.262	-100.231	-44.819	7.338
30	-5.99	-5.99	0	-1.219	-10.941	-35.959	0.141	-0.195	-101.057	-46.9	9.724
31	-5.984	-5.984	-0.021	-0.893	-18.977	-30.985	0.141	-0.188	-102.639	-49.962	13.502
32	-5.964	-5.964	-0.198	-0.398	-25.163	-21.067	0.099	-0.147	-99.471	-46.231	11.043
33	-5.954	-5.954	-0.064	-0.894	-13.92	-37.314	0.141	-0.441	-104.772	-51.233	10.238
34	-5.933	-5.933	-0.022	-0.819	-12.737	-36.172	0.141	-0.371	-98.201	-48.909	18.313
35	-5.919	-5.919	-0.029	-0.457	-18.585	-27.35	0.141	-0.174	-99.062	-45.934	11.311
36	-5.914	-5.914	0	-0.738	-15.854	-32.528	0.141	-0.3	-93.246	-48.382	18.923
37	-5.898	-5.898	-0.004	-0.421	-17.322	-28.68	0.099	-0.176	-97.038	-46.002	15.076
38	-5.847	-5.847	0	-1.255	-7.685	-40.08	0.141	-0.197	-101.3	-47.765	8.149
39	-5.837	-5.837	0	-0.831	-13.034	-43.127	0.141	-0.441	-110.333	-56.161	6.676
40	-5.834	-5.834	-0.108	-0.343	-25.031	-21.059	0.099	-0.152	-98.179	-46.09	10.786
41	-5.809	-5.809	-0.059	-0.903	-10.904	-42.996	0.141	-0.443	-107.19	-53.9	7.876
42	-5.796	-5.796	-0.033	-0.992	-8.918	-36.364	0.141	-0.241	-77.971	-45.282	41.099
43	-5.794	-5.794	-0.066	-0.507	-15.564	-27.155	0.141	-0.21	-92.222	-42.718	16.061

44	-5.772	-5.772	-0.024	-0.445	-18.042	-29.287	0.141	-0.168	-97.349	-47.33	14.604
45	-5.73	-5.73	0	-1.098	-6.946	-36.833	0.141	-0.335	-92.967	-43.779	14.53
46	-5.721	-5.721	-0.143	-0.608	-22.322	-24.671	0.141	-0.204	-100.075	-46.993	7.453
47	-5.712	-5.712	-0.125	-0.275	-26.39	-18.601	0.099	-0.155	-95.255	-44.991	12.028
48	-5.712	-5.712	0	-0.65	-7.646	-34.796	0.099	-0.248	-92.294	-42.442	13.734
49	-5.711	-5.711	-0.062	-0.689	-16.741	-26.676	0.141	-0.264	-89.5	-43.417	12.748
50	-5.709	-5.709	-0.027	-0.462	-15.616	-26.435	0.141	-0.161	-92.831	-42.051	11.82
51	-5.686	-5.686	0	-0.843	-10.015	-31.367	0.141	-0.259	-92.82	-41.382	9.159
52	-5.662	-5.662	0	-0.39	-16.87	-29.812	0.141	-0.195	-93.603	-46.682	18.61
53	-5.646	-5.646	-0.032	-0.34	-17.773	-29.409	0.141	-0.152	-96.285	-47.182	13.931
54	-5.645	-5.645	-0.031	-0.2	-21.351	-28.382	0.141	-0.104	-97.917	-49.733	14.857
55	-5.634	-5.634	-0.018	-0.45	-18.028	-25.715	0.141	-0.173	-92.541	-43.743	14.303
56	-5.633	-5.633	-0.031	-0.261	-18.061	-27.109	0.141	-0.134	-94.487	-45.169	13.249
57	-5.611	-5.611	-0.03	-0.266	-17.09	-27.556	0.141	-0.14	-93.921	-44.645	12.887
58	-5.596	-5.596	-0.028	-0.233	-20.92	-25.344	0.141	-0.136	-95.157	-46.264	13.364
59	-5.591	-5.591	-0.008	-0.4	-18.734	-25.538	0.141	-0.182	-90.163	-44.272	19.262
60	-5.548	-5.548	-0.02	-0.58	-9.68	-25.599	0.099	-0.193	-80.819	-35.279	19.111
61	-5.639	-5.639	-0.004	-0.744	-17.95	-25.565	0.141	-0.19	-91.997	-43.514	15.024
62	-5.541	-5.541	-0.015	-0.237	-18.034	-28.41	0.141	-0.115	-89.555	-46.445	23.834
63	-5.472	-5.472	0	-0.996	-8.304	-31.54	0.141	-0.202	-86.735	-39.843	14.033
64	-5.466	-5.466	0	-0.445	-18.223	-24.493	0.141	-0.202	-83.301	-42.716	27.39
65	-5.448	-5.448	-0.006	-0.557	-18.354	-25.6	0.141	-0.191	-90.019	-43.953	16.071
66	-5.438	-5.438	0	-0.547	-4.973	-32.38	0.099	-0.211	-87.783	-37.352	6.299
67	-5.435	-5.435	0	-0.823	-8.211	-39.08	0.141	-0.158	-92.152	-47.291	17.373
68	-5.433	-5.433	-0.028	-0.464	-16.701	-24.123	0.141	-0.169	-87.432	-40.824	14.684
69	-5.377	-5.377	-0.01	-0.633	-14.681	-26.69	0.141	-0.141	-72.456	-41.371	36.613
70	-5.338	-5.338	-0.024	-0.267	-18.775	-24.604	0.141	-0.146	-89.415	-43.379	13.938
71	-5.322	-5.322	-0.048	-0.309	-18.471	-29.428	0.141	-0.183	-95.162	-47.899	10.341
72	-5.195	-5.195	0	-0.709	-3.663	-29.766	0.141	-0.22	-78.253	-33.429	12.726
73	-5.177	-5.177	0	-0.763	-1.099	-30.788	0.141	-0.276	-71.706	-31.888	22.375
74	-5.174	-5.174	-0.021	-0.455	-12.15	-27.463	0.141	-0.232	-84.88	-39.613	11.411
75	-5.149	-5.149	-0.002	-0.632	-18.805	-23.25	0.141	-0.188	-87.543	-42.055	11.287
76	-5.143	-5.143	0	-0.49	-8.775	-27.96	0.141	-0.356	-78.15	-36.735	18.509
77	-5.135	-5.135	0	-0.691	2.029	-28.601	0.141	-0.236	-65.912	-26.572	15.648
78	-5.111	-5.111	-0.035	-0.2	-19.89	-23.657	0.141	-0.098	-86.532	-43.547	14.742
79	-5.065	-5.065	0	-0.537	0.225	-29.239	0.141	-0.232	-67.696	-29.014	15.693
80	-4.806	-4.806	-0.002	-0.599	-18.703	-21.2	0.141	-0.188	-80.944	-39.903	13.368
81	-4.601	-4.601	-0.009	-0.094	-21.363	-20.591	0.141	-0.095	-76.452	-41.954	22.394
82	-4.414	-4.414	0	-0.267	-2.37	-26.385	0.141	-0.212	-65.056	-28.755	14.38
83	-4.41	-4.41	0	-0.389	0.646	-25.887	0.141	-0.312	-46.539	-25.241	38.417
84	-4.298	-4.298	-0.007	-0.273	-12.395	-22.79	0.141	-0.103	-62.16	-35.186	25.005
85	-4.267	-4.267	0	-0.405	-18.993	-17.935	0.141	-0.139	-69.503	-36.928	18.933
86	-4.015	-4.015	-0.105	-0.431	-28.38	-13.999	0.141	-0.098	-74.695	-42.379	9.121
87	-3.797	-3.797	-0.005	-0.205	-17.634	-17.888	0.141	-0.097	-65.32	-35.522	10.15
88	-3.772	-3.772	-0.376	-0.418	-26.892	-11.694	0.141	-0.004	-70.667	-38.586	5.125
89	-3.76	-3.76	-0.017	-0.177	-22.789	-16.253	0.141	-0.102	-67.419	-39.042	12.315
90	-3.642	-3.642	-0.559	-0.16	-32.391	-6.083	0.141	-0.015	-66.533	-38.474	10.797
91	-3.383	-3.383	0	-0.482	-0.874	-19.091	0.141	-0.136	-41.994	-19.965	18.096
92	-3.274	-3.274	-0.076	-0.022	-13.397	-16.853	0.141	-0.12	-59.213	-30.249	6.588
93	-3.172	-3.172	-0.002	-0.16	-11.026	-16.344	0.141	-0.083	-52.724	-27.37	7.574

94	-3.035	-3.035	-0.319	-0.16	-29.08	-5.164	0.141	-0.002	-58.197	-34.244	7.845
95	-3.028	-3.028	-0.125	-0.292	-22.485	-10.231	0.141	-0.083	-54.591	-32.716	11.872
96	-2.923	-2.923	-0.063	0	-18.467	-13.806	0.141	-0.006	-57.403	-32.273	7.327
97	-2.879	-2.879	-0.782	0	-32.202	-3.199	0.099	-0.001	-59.138	-35.401	5.425
98	-2.787	-2.787	-0.321	-0.344	-28.075	-3.764	0.141	-0.046	-52.929	-31.839	9.015
99	-2.686	-2.686	-0.579	0	-30.979	-3.635	0.099	-0.001	-55.238	-34.614	8.096
100	-2.685	-2.685	-0.573	-0.135	-27.598	-4.565	0.141	-0.002	-51.879	-32.163	9.899
101	-2.683	-2.683	-0.68	0	-30.974	-3.047	0.099	-0.001	-54.912	-34.02	7.51
102	-2.619	-2.619	-0.048	-0.048	-17.616	-11.607	0.141	-0.02	-48.662	-29.223	9.241
103	-2.564	-2.564	-0.444	-0.143	-28.513	-3.782	0.141	0	-51.41	-32.295	8.877
104	-2.546	-2.546	-0.562	0	-26.608	-4.523	0.099	-0.021	-50.686	-31.132	7.671
105	-2.453	-2.453	-0.644	-0.16	-24.023	-3.033	0.141	0	-41.962	-27.056	15.251
106	-2.446	-2.446	-0.477	-0.053	-28.223	-2.616	0.141	0	-48.007	-30.839	10.597
107	-2.437	-2.437	-0.538	-0.149	-25.338	-2.969	0.141	-0.061	-44.112	-28.307	13.17
108	-2.426	-2.426	-1.006	-0.117	-27.566	-0.329	0.141	-0.002	-44.039	-27.895	12.272
109	-2.363	-2.363	-0.584	0	-26.327	-3.275	0.099	-0.023	-47.236	-29.602	8.138
110	-2.362	-2.362	-0.213	0	-23.558	-6.488	0.141	0	-47.066	-30.046	9.346
111	-2.151	-2.151	-0.057	0	-14.949	-8.498	0.141	-0.062	-39.785	-23.447	6.834
112	-2.126	-2.126	-0.065	0	-15.732	-7.976	0.141	-0.069	-38.416	-23.709	9.635
113	-2.103	-2.103	-0.392	-0.16	-25.325	-1.272	0.141	-0.063	-42.735	-26.597	6.36
114	-1.984	-1.984	-0.201	-0.003	-23.514	-3.645	0.141	0	-39.254	-27.159	12.267
115	-1.981	-1.981	-0.203	-0.019	-24.94	-3.35	0.141	-0.001	-40.481	-28.29	12.015
116	-1.966	-1.966	-0.288	0	-25.321	-2.45	0.141	-0.002	-42.976	-27.771	5.714
117	-1.933	-1.933	-0.208	-0.059	-21.848	-3.775	0.141	0	-37.325	-25.623	12.112
118	-1.547	-1.547	-0.297	0	-21.322	-0.605	0.141	0	-31.039	-21.927	10.198

Compound **8**

	docking score	glide gscore	glide lipo	glide hbond	glide evdw	glide ecoul	glide erotb	glide esite	glide emodel	glide energy	glide einterna
1	-8.659	-8.659	-0.174	-1.039	-22.023	-27.597	0.125	-0.316	-113.507	-49.621	18.351
2	-8.32	-8.32	-0.159	-1.106	-24.065	-34.72	0.125	-0.347	-117.447	-58.785	23.076
3	-8.097	-8.097	-0.018	-1.008	-16.374	-31.787	0.125	-0.254	-110.308	-48.161	12.353
4	-8.068	-8.068	-0.024	-0.973	-16.4	-31.321	0.125	-0.255	-108.999	-47.721	13.594
5	-8.037	-8.037	-0.013	-1.056	-14.421	-32.594	0.125	-0.25	-108.151	-47.015	13.363
6	-8.034	-8.034	-0.02	-1.009	-14.707	-35.111	0.125	-0.255	-108.833	-49.818	17.548
7	-8.017	-8.017	-0.018	-1.022	-14.551	-34.56	0.125	-0.252	-110.712	-49.111	12.093
8	-7.729	-7.729	0	-1.232	-12.618	-33.142	0.125	-0.4	-112.92	-45.76	9.251
9	-7.63	-7.63	-0.004	-0.705	-13.497	-30.715	0.125	-0.24	-100.84	-44.211	15.506
10	-7.448	-7.448	-0.016	-0.977	-14.735	-32.755	0.125	-0.251	-104.709	-47.49	11.261
11	-7.394	-7.394	-0.018	-0.999	-12.9	-36.174	0.125	-0.265	-105.091	-49.075	12.753
12	-7.353	-7.353	-0.012	-0.983	-12.839	-34.65	0.125	-0.249	-103.416	-47.488	12.234
13	-7.309	-7.309	-0.003	-0.961	-12.489	-35.354	0.125	-0.254	-103.07	-47.843	12.894
14	-7.232	-7.232	0	-0.883	-12.914	-32.52	0.125	-0.234	-99.82	-45.433	13.269
15	-7.144	-7.144	-0.168	-0.547	-19.554	-28.624	0.125	-0.188	-100.221	-48.178	16.484
16	-7.07	-7.07	-0.018	-0.764	-13.516	-33.671	0.125	-0.389	-105.848	-47.187	14.017
17	-6.996	-6.996	-0.21	-1.342	-25.074	-46.485	0.178	-0.362	-121.965	-71.56	29.141
18	-6.351	-6.351	-0.091	-0.724	-16.777	-27.282	0.125	-0.267	-90.061	-44.059	15.177
19	-6.152	-6.152	0	-0.945	-16.358	-39.199	0.178	-0.266	-104.174	-55.557	6.592
20	-6.069	-6.069	-0.052	-0.903	-17.269	-28.444	0.178	-0.204	-88.287	-45.712	17.269
21	-6.036	-6.036	-0.089	-0.902	-20.952	-31.954	0.178	-0.176	-101.737	-52.905	14.468
22	-6.01	-6.01	-0.179	-0.682	-18.234	-23.578	0.125	-0.269	-86.869	-41.812	11.317
23	-6.007	-6.007	-0.117	-0.748	-18.859	-23.333	0.125	-0.266	-86.736	-42.193	12.282
24	-5.972	-5.972	-0.132	-0.846	-20.646	-31.763	0.178	-0.14	-101.602	-52.409	12.556
25	-5.955	-5.955	-0.284	-0.321	-26.548	-19.607	0.125	-0.144	-89.49	-46.156	13.82
26	-5.915	-5.915	-0.119	-0.842	-19.191	-35.942	0.178	-0.174	-102.785	-55.132	14.576
27	-5.879	-5.879	-0.006	-1.142	-14.636	-36.286	0.178	-0.177	-98.735	-50.921	13.578
28	-5.783	-5.783	-0.168	-0.273	-23.877	-20.403	0.125	-0.156	-86.046	-44.279	14.062
29	-5.737	-5.737	-0.117	-0.472	-19.896	-26.86	0.178	-0.158	-93.186	-46.756	13.716
30	-5.697	-5.697	-0.146	-1.096	-14.331	-23.652	0.178	-0.143	-86.133	-37.984	9.523
31	-5.608	-5.608	-0.29	-0.373	-23.321	-23.69	0.178	-0.141	-87.744	-47.011	13.17
32	-5.419	-5.419	0	-0.848	-11.937	-26.762	0.178	-0.153	-84.231	-38.699	9.6
33	-5.38	-5.38	-0.285	-0.361	-23.409	-22.326	0.178	-0.141	-85.198	-45.734	11.866
34	-5.369	-5.369	-0.123	-0.591	-16.043	-25.406	0.178	-0.221	-84.602	-41.449	13.434
35	-5.368	-5.368	-0.246	-0.462	-14.059	-30.896	0.178	-0.136	-87.516	-44.955	14.589
36	-5.305	-5.305	-0.015	-0.496	-11.348	-30.031	0.178	-0.404	-81.933	-41.379	8.411
37	-4.935	-4.935	-0.011	-0.863	-15.371	-22.276	0.178	-0.129	-77.16	-37.646	12.641
38	-4.824	-4.824	-1.178	-0.406	-35.806	-7.591	0.178	-0.007	-76.281	-43.397	15.635
39	-4.809	-4.809	0	-0.432	-15.157	-20.649	0.125	-0.117	-71.697	-35.806	9.375
40	-4.793	-4.793	-0.047	-0.45	-18.187	-19.181	0.125	-0.105	-73.047	-37.367	9.522
41	-4.642	-4.642	-0.019	-0.528	-14.588	-18.821	0.125	-0.137	-66.132	-33.41	12.896
42	-4.436	-4.436	-0.185	-0.332	-14.358	-18.67	0.178	-0.203	-65.388	-33.027	10.139
43	-4.381	-4.381	-0.018	-0.454	-11.894	-22.178	0.178	-0.166	-68.948	-34.072	11.638

44	-4.323	-4.323	-0.123	-0.408	-16.982	-19.781	0.178	-0.154	-71.123	-36.764	4.239
45	-4.218	-4.218	-0.107	-0.435	-17.881	-15.114	0.125	-0.109	-69.558	-32.996	5.225
46	-4.174	-4.174	-0.294	-0.153	-27.862	-15.713	0.178	-0.065	-73.524	-43.576	10.536
47	-4.076	-4.076	0	-0.318	-6.904	-23.386	0.178	-0.084	-60.795	-30.29	14.712
48	-3.992	-3.992	0	-0.309	-7.79	-22.48	0.178	-0.1	-61.963	-30.27	10.775
49	-3.961	-3.961	-0.146	-0.435	-22.732	-15.079	0.178	-0.159	-66.115	-37.811	16.964
50	-3.86	-3.86	-0.71	-0.16	-29.346	-8.438	0.178	0	-71.757	-37.784	3.75
51	-3.476	-3.476	-0.176	-0.057	-23.567	-14.226	0.178	-0.053	-64.794	-37.794	10.568
52	-3.456	-3.456	-0.651	-0.16	-31.997	-5.581	0.178	0	-68.456	-37.577	2.451
53	-3.447	-3.447	-0.652	0	-31.243	-8.194	0.178	0	-64.934	-39.437	7.177
54	-3.396	-3.396	-0.712	-0.147	-30.031	-7.519	0.178	0	-62.257	-37.551	7.895
55	-3.377	-3.377	-0.556	-0.151	-26.761	-9.318	0.178	0	-61.083	-36.08	6.979
56	-3.363	-3.363	-0.849	-0.133	-32.665	-3.848	0.178	-0.011	-60.761	-36.513	8.254
57	-3.336	-3.336	-0.673	-0.148	-29.012	-7.519	0.178	0	-59.568	-36.531	10.218
58	-3.228	-3.228	-0.001	-0.456	-10.09	-15.405	0.178	-0.133	-48.843	-25.495	13.259
59	-3.153	-3.153	-0.09	-0.485	-18.207	-12.122	0.178	-0.027	-55.387	-30.329	8.45
60	-3.087	-3.087	-0.22	-0.2	-22.83	-11.242	0.178	-0.011	-53.888	-34.072	12.46
61	-3.036	-3.036	-0.538	0	-25.43	-8.388	0.178	-0.003	-53.117	-33.818	12.639
62	-3.033	-3.033	-0.69	-0.16	-29.453	-4.375	0.178	0	-53.377	-33.828	12.083
63	-3.016	-3.016	-0.044	-0.356	-18.415	-11.993	0.178	-0.07	-55.022	-30.407	6.801
64	-2.845	-2.845	0	-0.26	-11.588	-13.769	0.178	-0.119	-45.155	-25.357	13.26
65	-2.769	-2.769	-0.156	-0.311	-16.505	-10.308	0.178	-0.109	-47.861	-26.813	9.345
66	-2.752	-2.752	-0.016	-0.183	-10.986	-13.786	0.178	-0.114	-45.67	-24.771	9.329
67	-2.745	-2.745	-0.037	-0.182	-17.825	-11.887	0.178	-0.03	-50.09	-29.712	10.231
68	-2.688	-2.688	-0.086	-0.069	-18.964	-11.534	0.178	-0.033	-52.249	-30.498	6.438
69	-2.655	-2.655	0	-0.317	-16.119	-11.048	0.178	-0.05	-46.745	-27.167	10.161
70	-2.634	-2.634	0	-0.059	-17.631	-11.915	0.178	-0.085	-49.284	-29.545	9.453
71	-2.621	-2.621	-0.031	-0.2	-16.497	-11.334	0.178	-0.044	-49.209	-27.831	5.938
72	-2.428	-2.428	-0.127	-0.055	-18.492	-9.355	0.178	-0.08	-44.586	-27.847	11.628
73	-2.415	-2.415	0	-0.088	-12.527	-11.955	0.178	-0.086	-42.335	-24.483	9.166
74	-2.358	-2.358	-0.053	-0.283	-22.707	-6.886	0.178	-0.018	-48.55	-29.593	5.883
75	-2.329	-2.329	-0.042	0	-14.479	-11.398	0.178	-0.031	-40.529	-25.877	13.957
76	-2.32	-2.32	-0.04	-0.248	-23.065	-6.835	0.178	-0.017	-48.638	-29.9	5.621
77	-1.751	-1.751	-0.002	-0.019	-15.592	-7.046	0.178	-0.072	-30.918	-22.638	15.964

Glide emodel. Combines the energy of **glide score** (calculated from **glide energy**), which is the binding affinity predicted by glide score, and in the case of flexible docking also the internal strain energy (**glide internal**) that is used to model the potential used to direct the conformational-search algorithm.

Glide evdw: Van der Waals energy. This term is calculated with reduced net ionic charges on groups with formal charges, such as metals, carboxylates, and guanidiniums.

Glide ecoul: Coulomb energy. This term is calculated with reduced net ionic charges on groups with formal charges, such as metals, carboxylates, and guanidiniums.

Glide lipo: Lipophilic term, which is a pairwise term in SP but is derived from the hydrophobic grid potential for XP. Rewards favourable hydrophobic interactions.

Glide hbond: Hydrogen-bonding term. This term is separated into differently weighted components that depend on whether the donor and acceptor are neutral, one is neutral and the other is charged, or both are charged.

Gide erotb: Penalty for freezing rotatable bonds.

Glide esite: Site Polar interactions in the active site. Polar but non-hydrogen-bonding atoms in a hydrophobic region are rewarded.

1.- Friesner, R. A.; Banks, J. L.; Murphy, R. B.; Halgren, T. A.; Klicic, J. J.; Mainz, D. T.; Repasky, M. P.; Knoll, E. H.; Shelley, M.; Perry, J. K.; Shaw, D. E.; Francis, P.; Shenkin, P. S. Glide: A new approach for rapid, accurate docking and scoring. 1. Method and assessment of docking accuracy. *J. Med. Chem.* **2004,** *47* (7), 1739-1749.
2.- Halgren, T. A.; Murphy, R. B.; Friesner, R. A.; Beard, H. S.; Frye, L. L.; Pollard, W. T.; Banks, J. L. Glide: A New Approach for Rapid, Accurate Docking and Scoring. 2. Enrichment Factors in Database Screening. *J. Med. Chem.* **2004,** *47* (7), 1750-1759.
